# Low Connectivity between Mediterranean Marine Protected Areas: A Biophysical Modeling Approach for the Dusky Grouper *Epinephelus marginatus*

**DOI:** 10.1371/journal.pone.0068564

**Published:** 2013-07-08

**Authors:** Marco Andrello, David Mouillot, Jonathan Beuvier, Camille Albouy, Wilfried Thuiller, Stéphanie Manel

**Affiliations:** 1 UMR 151 - Laboratoire Population Environnement et Développement, Institut de Recherche pour le Développement - Université Aix-Marseille, Marseille, France; 2 UMR 5553 - Laboratoire d’Écologie Alpine, Centre National de la Recherche Scientifique - Université Grenoble 1, Grenoble, France; 3 UMR 5119 - Écologie des Systèmes marins côtiers, Université Montpellier 2, Montpellier, France; 4 ARC Centre of Excellence for Coral Reef Studies, James Cook University, Townsville, Australia; 5 Mercator Océan, Ramonville-Saint-Agne, France; 6 UMR 3589 - Groupe d’Étude de l’Atmosphère Météorologique, Centre National de Recherches Météorologiques - Centre National de la Recherche Scientifique - Météo-France, Toulouse, France; 7 Département de Biologie, Chimie et Géographie, Université du Québec à Rimouski, Rimouski, Canada; 8 UMR 5120 - Botanique et Bioinformatique de l’Architecture des Plantes, Montpellier, France; Bangor University, United Kingdom

## Abstract

Marine protected areas (MPAs) are major tools to protect biodiversity and sustain fisheries. For species with a sedentary adult phase and a dispersive larval phase, the effectiveness of MPA networks for population persistence depends on connectivity through larval dispersal. However, connectivity patterns between MPAs remain largely unknown at large spatial scales. Here, we used a biophysical model to evaluate connectivity between MPAs in the Mediterranean Sea, a region of extremely rich biodiversity that is currently protected by a system of approximately a hundred MPAs. The model was parameterized according to the dispersal capacity of the dusky grouper *Epinephelus marginatus*, an archetypal conservation-dependent species, with high economic importance and emblematic in the Mediterranean. Using various connectivity metrics and graph theory, we showed that Mediterranean MPAs are far from constituting a true, well-connected network. On average, each MPA was directly connected to four others and MPAs were clustered into several groups. Two MPAs (one in the Balearic Islands and one in Sardinia) emerged as crucial nodes for ensuring multi-generational connectivity. The high heterogeneity of MPA distribution, with low density in the South-Eastern Mediterranean, coupled with a mean dispersal distance of 120 km, leaves about 20% of the continental shelf without any larval supply. This low connectivity, here demonstrated for a major Mediterranean species, poses new challenges for the creation of a future Mediterranean network of well-connected MPAs providing recruitment to the whole continental shelf. This issue is even more critical given that the expected reduction of pelagic larval duration following sea temperature rise will likely decrease connectivity even more.

## Introduction

Most of world’s marine ecosystems are under unprecedented overfishing levels [1]. As human populations continue to grow, a major challenge is to counteract the depletion of fish stocks, which largely relies on how well they are rebuilt and managed. Marine protected areas (MPAs) are indisputably the flagship tools to protect biodiversity and fish populations worldwide as well as to restore overexploited stocks [2]–[6].

The effectiveness of MPAs for protecting exploited species depends, at least partly, on connectivity through its effects on population dynamics and genetics [6]–[13]. The species that benefit most from MPAs are sessile and territorial organisms [2], [14], [15], for which connections between local populations are maintained through larval dispersal only. Connectivity between MPAs is therefore dependent on the patterns and strength of larval dispersal and determines their success as tools to protect biodiversity and enhance fisheries through recruitment supply beyond their boundaries.

Given recent methodological advances in larval tagging [16], [17], analysis of otolith microchemistry [10], [18], genetic parentage analysis [19]–[21] assignment tests [22], [23] and biophysical modeling [24], [25], dispersal trajectories of larvae are better known. All these methods differ in the spatial and temporal scales of applicability and have different strengths and weaknesses. Larval tagging, parentage analysis and otolith microchemistry have proved effective in resolving marine connectivity over relatively short spatial and temporal scales. However, since they require intensive sampling, they are relatively costly. Biophysical models offer the possibility to track virtual individuals over longer spatial and temporal scales, but require knowledge of numerous biological and physical parameters and validation with empirical data. These approaches are therefore complementary and should be used together when possible to measure the consistency of their outputs [8]. Nevertheless, the biophysical modeling approach can provide a first assessment of connectivity at large spatial and temporal scales, for which the other methods are still inapplicable, or help design an optimal sampling strategy [26].

**Figure 1:**
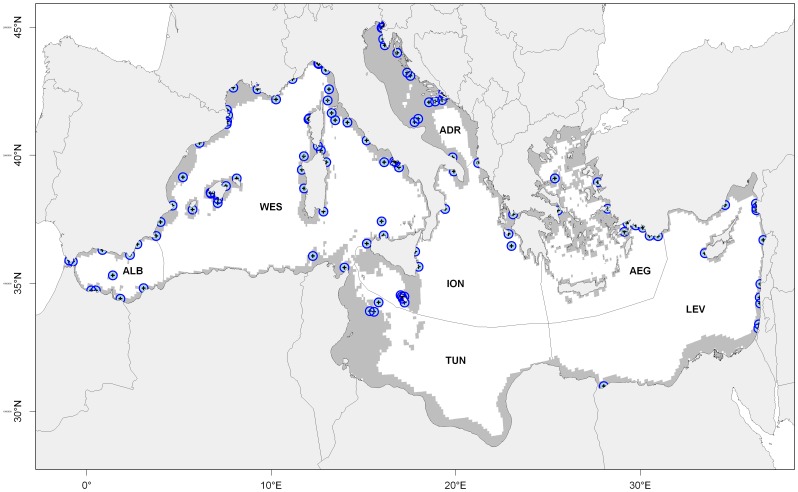
The locations of the 99 MPAs. The size of blue circles does not reflect MPA size. Letters within circles denote ecoregions [108]: ALB, Alboran Sea; WEST, Western Mediterranean; TUN, Tunisian Plateau and Gulf of Sidra; ION, Ionian Sea; ADR, Adriatic Sea; AEG, Aegean Sea; LEV, Levantine Sea.

The Mediterranean Sea harbors more than 600 fish species with a high fraction of endemism [27] and experiences unprecedented levels of human pressure from fishing, exotic species, and pollution [28]. The Mediterranean Sea benefits from the presence of approximately a hundred MPAs, mainly concentrated in its Northern coastal areas (Figure 1) [28]–[30]. Previous studies showed that fishing restrictions in Mediterranean MPAs have positively affected the density, size, biomass, and diversity of exploited species [4]–[6]. However, the connectivity among MPAs and their ability to provide recruitment benefits beyond their boundaries remain under scrutiny [6], [10]. This knowledge is crucial since MPA networks need to ensure the persistence of target species and to maintain fisheries yield over large scales.

Here, we implemented a biophysical model to estimate recruitment within Mediterranean MPAs, connectivity among them and larval export to fished areas for an overexploited species: the dusky grouper *Epinephelus marginatus* (Lowe, 1834). *Epinephelus marginatus* is sedentary and territorial in its adult phase [31]–[33] and represents the archetypical fish benefiting from MPAs, since it was heavily targeted for food consumption, particularly by spearfishing, and was nearly eradicated from the Northern coast of the Mediterranean [34] until it was listed as endangered by the International Union for the Conservation of Nature [35]. Due to its slow growth and late maturation, *E. marginatus* should be considered as a “conservation-dependent” species, a species for which the role of MPAs is critical for maintaining a viable population [32], [36], [37]. By considering that MPAs concentrate *E. marginatus* adults in the Mediterranean Sea [38], we assessed the patterns of recruitment and connectivity among MPAs and whether larval export from MPAs benefits exploited areas over the entire continental shelf of the Mediterranean Sea. To this aim, we used graph theory [39]–[41] to describe and analyze connectivity patterns among MPAs and to rank MPAs according to their importance for system connectivity. More precisely, we used i) neighborhood size, which measures the number of MPAs that are directly connected; ii) cluster identification, which extends the analysis of connectivity beyond direct connections to identify groups of MPAs that can exchange genes through multi-step connections across generations; and iii) betweenness centrality, to identify which MPAs act as gateways or obligate routes during this multigenerational gene transfer.

## Materials and Methods

### Spatial Distribution of MPAs

The Mediterranean Sea contains approximately a hundred coastal MPAs of various sizes [29]. MPAs were digitized on the basis of MedPan data (http://www.medpan.org/) as well as from maps, polygons, and GPS coordinates provided by managers [30] (Figure 1). Some MPAs are subdivided into non-contiguous zones (e.g. the Tuscan Archipelago MPA consists of five separate zones around the islands of Giannutri, Montecristo, Pianosa, Capraia and Gorgona): each zone was treated as a single MPA, providing a total of 115 MPAs (Table S1). These MPAs implement various levels of fishing protection and enforce regulation with varying effectiveness; for these reasons, Mediterranean MPAs do not systematically have positive effects on biodiversity and fish biomass [3], [5]. At the time of our study, a comprehensive list of MPAs and associated levels of protection and regulation enforcement was not available. We thus considered an unrealistic and optimistic scenario where all MPAs enforce fishing restrictions and maintain dusky grouper populations. The results of our study should therefore be considered as potential connectivity patterns on the basis of the formally existing MPAs.

### Biological Features of E. marginatus

The dusky grouper *E. marginatus* (Pisces, Serranidae) is a rocky-bottom-associated species that inhabits coastal reefs from shallow water out to a depth of 50 m along all Mediterranean coasts [42], [43]. Observations of the species are very frequent in the MPAs of North-Western Mediterranean [33], [44], but observations in the Southern and Eastern Mediterranean are scarce and seldom reported. Similarly, the distribution and abundance of suitable habitats for groupers is poorly known in the South-Eastern Mediterranean. However, the distribution area of the species overlaps with all the MPAs and we considered them suitable places for harboring dusky grouper populations.

Adult dusky groupers are territorial, display strong site fidelity, and form small spawning aggregations in summer months [31]–[33], [44], [45]. Spawning events were seldom observed in the sea and were always concentrated in August [44], [45]. In absence of fishing, groupers can grow to large sizes above which reproductive females can change their sex and reproductive males can spawn. This makes MPAs suitable places for reproduction, but males are found also in fished areas and reproduction is possible if fishing activity is not too intense.

Data on the dispersal potential of pelagic larvae are scarce, but field observations suggest that larvae can remain up to 30 day in the planktonic stage [46] and for longer times (>40 days) when reared in the laboratory [47]–[50]. No data on larval behavior (active swimming, sensory ability, vertical migration) are available.

### Hydrodynamic Model

Three-dimensional sea current velocities were calculated with the NEMOMED12 model [51], [52]. NEMOMED12 is a Mediterranean Sea regional configuration of the ocean general circulation model NEMO [53]. NEMO resolves the primitive equations, with the spheric-earth approximation, the thin-shell approximation, the turbulent closure hypothesis, the Boussinesq hypothesis, the hydrostatic hypothesis and the incompressibility hypothesis. It uses finite differences on a C-type Arakawa grid.

The model has a horizontal resolution of 1/12^th^ degree, corresponding to a 6–8 km horizontal cell width; we thus consider this model as eddy-resolving, as the first Rossby radius of deformation ranges between 10 and 15 km in the main parts of the Mediterranean Sea [51]. However, a spatial resolution of 1/12^th^ degree might be too coarse to resolve small-scale circulation, particularly around complex topographical features such as headlands and semi-enclosed bays [54]. The model has 50 vertical layers, unevenly spaced from 1 m-thick at the surface to 450 m-thick at the bottom, with 35 levels in the first 1000 m. The bathymetry comes from the GEBCO-08 database (http://gebco.net version 20081212), the MEDIMAP bathymetry [55] and the Ifremer bathymetry of the Gulf of Lions [56]. The original bathymetry product has a resolution of 1/120^th^ degree and is interpolated on the NEMOMED12 grid. We use a partial cell parameterization, *i.e.* the bottom layer thickness is varying to fit the real bathymetry.

We only report the main numerical choices and parameterisations. A time step of 12 minutes was used. The horizontal eddy diffusivity coefficient was set to 60 m^2^ s^-1^ for the tracers (temperature, salinity) using a laplacian operator (the diffusion is applied along iso-neutral surfaces for the tracers) and the horizontal viscosity coefficient was set to −1.25⋅10^10^ m^4^ s^−2^ for the dynamics (velocity) using of a biharmonic operator. The TVD (Total Variance Dissipation) scheme was used for the tracer advection and the EEN (Energy and ENstrophy conservative) scheme was used for the momentum advection [57], [58]. A 1.5 turbulent closure scheme was used for the vertical eddy diffusivity [59], with an enhancement of the vertical diffusivity coefficient up to 10 m^2^ s^−1^ in case of unstable stratification. A no-slip lateral boundary condition was used.

The model was forced with the atmospheric wind stress, total and solar heat fluxes, total freshwater flux (evaporation minus precipitation), and river discharge (33 main rivers mouths plus a coastal runoff). These atmospheric fields came from the ARPERA dataset [60], which has a resolution of 50 km above the Mediterranean area. NEMOMED12 returns the three-dimensional velocity, potential temperature, salinity and water potential density fields, and the sea surface elevation, from October 1st 1998 to December 31st 2008.

NEMOMED12, under this configuration, was used to study the spreading of deep water masses in the Western Mediterranean [51]. It has also shown its ability to well reproduce the interannual variability and extreme events of thermohaline characteristics in the whole Mediterranean in long-term simulations [52], [61], with respects to interannual gridded climatologies [62], [63]. As far as surface circulation is concerned, comparisons with altimetry products indicate that NEMOMED12 reproduces the observed circulation patterns in the Mediterranean [61] as well as high eddy activities at meso-scale [51].

Because of heat and water exchanges with the Atlantic Ocean and with the atmosphere, the Mediterranean Sea is a concentration basin, which transforms the warm and fresh Atlantic Water into colder and saltier Mediterranean water masses. The Atlantic Water enters the Mediterranean at the Strait of Gibraltar in the surface layer and flows around the sea in a cyclonic path of more or less stable boundary currents, producing meanders and eddies [64]. The general surface circulation in the Mediterranean is strongly constrained by the wind [65] and by the complex bathymetry [66].

### Larval Dispersal Simulation

Daily outputs of zonal and meridional velocities from NEMOMED12 were used in this study to simulate passive larval dispersion using the software Ichthyop 3.1 [67]. As the tidal signal in the Mediterranean is relatively small, daily currents were considered appropriate inputs to Ichthyop and were obtained by averaging over the time-steps of NEMOMED12. Thus, circulations acting at higher frequencies are not considered. In the simulation, we released larvae every three days from the 1^st^ of August until the 28^th^ of August (ten release events), according to the spawning period of the dusky grouper observed in the North-Western Mediterranean [44]. The actual spawning period may vary across the distribution area of the dusky grouper, but current knowledge is insufficient to model this variation. To test the effect of a variation in the spawning season on the results, we modeled larval dispersal using two alternative spawning months: July 1^st^–28^th^ and September 1^st^–28^th^. We ran 10 replicates for each month and compared the results using ANOVA tests.

For a single year, therefore, there were 10 release events per MPA, each including 1000 larvae, which gives 10,000 larvae released per MPA per year. To smooth inter-annual variability due to sea currents, we pooled the larval dispersal simulations for five years. Thus, over the five years, this means that we used 50,000 larvae per MPA (5750000 larvae in total). This allowed us to detect connection probabilities as small as 0.00002. Larvae were released at 20 cm depth, because eggs were seen ascend to the surface immediately after spawning [45].

The time step of iteration was chosen so as to be lower than the ratio of cell size to maximum current velocity, so that larvae do not cross more than one cell boundary in a single time step. In our case, cell size was ∼6–8 km and maximum current velocity was ∼0.5 m s^−1^, giving a 12000–16000 s ratio, so we chose a 7200 s (2 h) time-step of iteration. Current velocities were tri-linearly interpolated to the position of each larva, each iteration, and the positions of larvae were recorded every 12 iterations (24 h). Advection was simulated using an Eulerian numerical scheme. Horizontal diffusion was applied via a random walk for individual larvae to account for sub-grid-scale hydrodynamics associated with coastal features (reefs, bays, gulfs, etc.) following Peliz et al. [68]. They suggest that floating velocity be calculated by adding a random component *u_r_*(*x*,*y*) to the horizontal velocity vector, with . Here, δ is a real uniform random number in the interval [−1, 1], Δ*t* is the time step of iteration and *K_h_* is the lagrangian horizontal diffusion coefficient 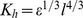, following Monin and Ozmidov [69], with ε = turbulent dissipation rate (m^2^ s^−3^) and *l* = length of the grid cell. Monin and Ozmidov suggested ε = 10^−9^. In Ichthyop, larvae intercepting the outer limits of the domain were considered lost; larvae reaching the sea surface were sent back into water; larvae floating in the bottom layers did not touch the seabed because the current velocity was zero at the vertical midpoint of the layer. Larvae reaching the interface between sea and land were retained in place (*standstill* option in Ichthyop).

The duration of larval transport (pelagic larval duration, PLD) was set to 30 days, but we explored values between 20 days and 40 days, reflecting the variation in PLD observed in laboratory studies and field observations [46]–[50]. This allowed us to account for the variability in PLD between individuals, to treat the uncertainty in PLD estimation from laboratory studies and field observations and to investigate the effects of decreased or increased PLD on connectivity. The effect of PLD on the results was tested using linear regression.

Larvae were subject to sea currents only (passive dispersal) without simulating active swimming or vertical migration. Since vertical behaviour can significantly affect larval dispersal [70], we ran a sensitivity analysis to compare the results obtained with vertical migration to those obtained under the passive dispersal scenario. Since no data on vertical migration are available for dusky grouper larvae, we only built one scenario where larvae ascend to the surface at night and descend to lower depth at day following what has been observed on other species [71]. This type of diel vertical migration may constitute a predator avoidance mechanism or a metabolic advantage [72]. Specifically, the larvae were forced to ascend to 20 cm depth at 8pm and to descend to 50 m depth at 8am. We ran 10 replicates of each scenario and compared the results using Student’s *t* test, since replicates were normally distributed around their means and had homogenous variances.

### Analysis

Larval dispersal distance was measured as great-circle distance (the shortest distance between two points on a sphere) from the release point to the final point at the end of larval transport. Great-circle distances are likely smaller than oceanographic distances. We chose to use great-circle distances because they are more easily comparable to Euclidean distances between MPAs. Studies on MPAs usually employ Euclidean distances rather than oceanographic distances [73], [74], because they can be used to find the minimum distance between MPAs that allows for effective connectivity. In any case, the use of great-circle distances or oceanographic distances does not affect the estimation of connectivity and larval export.

The connection probability *c*(*i*,*j*) was the fraction of larvae originating in MPA *j* that ended up in MPA *i*. We defined the *connectivity matrix* **C** as the matrix formed by the connection probabilities *c(i,j)*. *Connectance* was defined as the fraction of connections with nonzero probability out of the total number of connections (*i.e.* the number of nonzero elements of **C** divided by the squared size of **C**). The *local retention fraction* was defined as the fraction of larvae released from a source MPA that settled back to that MPA, *lr*(*i*) = *c*(*i*,*i*). The *self-recruitment fraction* was defined as the fraction of total larval recruits to a MPA that originated from that MPA:
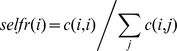



The *subsidy recruitment fraction* was the fraction of total larval recruits coming from other MPAs:
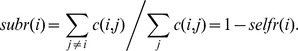



Larval export was illustrated graphically in maps of larval abundance at the end of the dispersal phase and quantitatively as fraction of continental shelf area not receiving larvae. These maps must be interpreted as potential larval export if larval production was constant across MPAs and absent outside MPAs. Larval production is likely very variable among MPAs, because of their differences in size, time since protection, fishing restrictions and regulation enforcement [3], [5]. Due to a lack of data, we can only account for MPA size. We thus also draw maps under the hypothesis that larval production is proportional to MPA size.

We used graph theory to quantify the connectivity within the network of MPAs [40], [75]. MPAs and larval trajectories defined nodes and edges of the graph, respectively. We calculated neighborhood sizes for each MPA, identified clusters (subgraphs) of connected MPAs and calculated the betweenness centrality of each MPA. The first-order neighborhood of a node (MPA) is composed by the nodes directly connected to it. As connections are directional, we calculated the *upstream neighborhood size* and the *downstream neighborhood size* for each MPA [40]. The upstream neighborhood of a MPA comprises all the MPAs that send larvae to that MPA through direct links. The downstream neighborhood of a MPA comprises all MPAs that receive larvae from that MPA through direct links.

Neighborhood sizes count first-order connections but a node can be connected to other nodes through multistep connections, *i.e.* connections that go through two or more nodes. A set of connected nodes form a *cluster* or subgraph. Specifically, nodes in a cluster are considered connected when there is a directed path connecting every pair of nodes in that cluster, *i.e.* it is possible to reach all nodes in the cluster starting from any node. This criterion is known as the ‘strong’ connectivity criterion and applies here because connections are directional. For comparison, we also identified clusters formed by nodes connected in a ‘weak’ sense: weak connectivity implies the existence of an undirected path between every pair of nodes in the cluster. The weak connectivity criterion is less stringent, because undirected paths do not imply the possibility of reaching every node starting from any other node in the cluster.

From the above definition of strong connectivity, it follows that when two nodes *k* and *l* are connected (i.e. part of the same cluster), there is at least one path going from *k* to *l*. If multiple paths exist, one can identify the shortest path connecting node *k* to node *l* as a path going through the minimum number of nodes regardless of the distance [39], [41]. Shortest paths are important because they are the fastest routes through which individuals or genes can spread from a node to all the other nodes in their cluster. The *betweenness centrality* metric measures the proportion of shortest paths between nodes that pass through a given node. Specifically, the *betweenness centrality* of node *i* is the proportion of shortest paths connecting vertices *k* and *l* that pass through node *i*, summed on all possible node pairs:
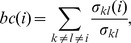
where *σ_kl_* is the number of shortest paths connecting nodes *k* and *l* and *σ_kl_* (*i*) is the number of those passing through the node *i*. Thus, betweenness centrality measures the importance of single nodes to act as corridors from spreading genes or individuals through the network [41] [76] and relates to the ‘stepping stone’ landscape ecology concept [39]. During this multigenerational transfer, nodes with high betweenness centrality are obligate “gateways” through which genes have to pass in order to spread to other nodes. Betweenness centrality allowed us to identify which nodes (MPAs) were the most important for multigenerational connectivity within the Mediterranean network.

Neighborhood sizes and betweenness centrality are therefore two complementary node-level metrics that quantify the connectivity of single MPAs in relation to their closest neighbors (neighborhood size) or to all nodes within their clusters (betweenness centrality).

Analyses were performed using the software R [77]. Connection probabilities and larval abundances were calculated from the positions of larvae in the last time step of the dispersal phase with the function *point.in.polygon* in the R package *sp* [78], without integration in time over a certain period (larval competency phase). Neighborhood sizes, number of strongly and weakly connected clusters and betweenness centrality were calculated with the R package *igraph* [79].

## Results

### Larval Dispersal Distances

**Figure 2:**
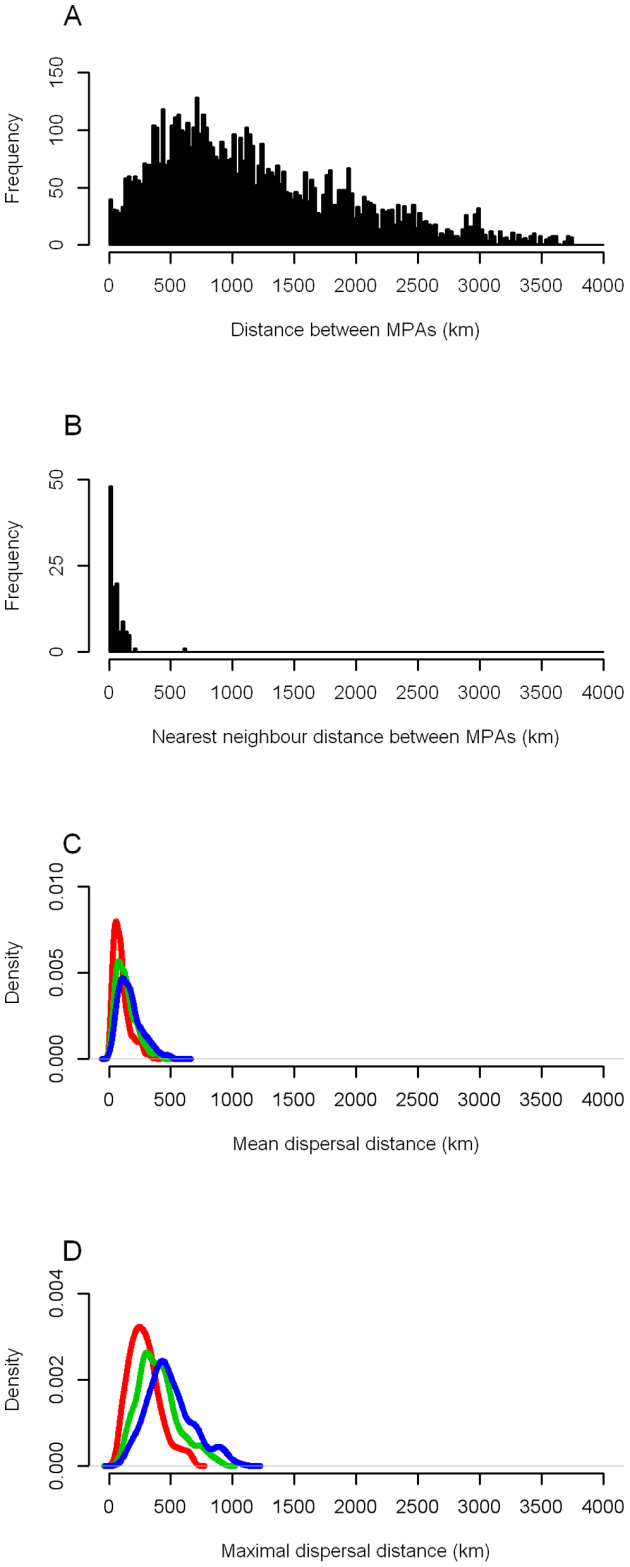
Frequency distributions of geographic distances. A, Between all pairs of MPAs; B, between each MPA and its nearest neighbors; C, mean and D, maximal distances of larval transport for each MPA for the three PLDs (red, 20 days; green, 30 days; blue, 40 days).h.

Pairwise distances between MPAs ranged from 1 km to 3748 km (median 1032 km, interquartile range 1117 km; Figure 2A), and the nearest neighbor distance between MPAs ranged from 1 km to 610 km (median 39 km, interquartile range 57 km; Figure 2B). 80% of MPAs had at least one neighboring MPA within 100 km, while the most isolated MPA (Sallum, Egypt) had a nearest neighbor more than 600 km away. Larval dispersal distances were shorter than pairwise distances between MPAs but longer than nearest neighbor distances. The mean dispersal distance ranged from 14 km to 522 km (median 120 km, interquartile range 102 km; Figure 2C) and the maximal dispersal distance ranged from 73 km to 906 km (median 377 km, interquartile range 200 km; Figure 2D). As a consequence, distant MPAs (>1000 km) cannot be directly connected, while neighboring MPAs have the potential for connectivity.

### Self-recruitment, Subsidy Recruitment and Local Retention

Local retention fraction was very low on average and highly variable among MPAs (median 0.00120, interquartile range 0.00584). Self-recruitment fractions were high (median 0.37, interquartile range 0.42) and continuously distributed in the full range of possible values (between 0 and 1). Six MPAs relied totally on self-recruitment (Debeli Rtic, Foca, Schinia-Marathona, Lara Toxeftra, Sallum and Porto Cesareo) while six others had zero self-recruitment (Isole Egadi, Port-Cros, Secche di Tor Paterno, Isole Pelagie-Lampione, Isole Tremiti-San Domino and Isole Tremiti-Pianosa) and relied only on subsidy recruitment (Figure S1).

### Connections

**Figure 3:**
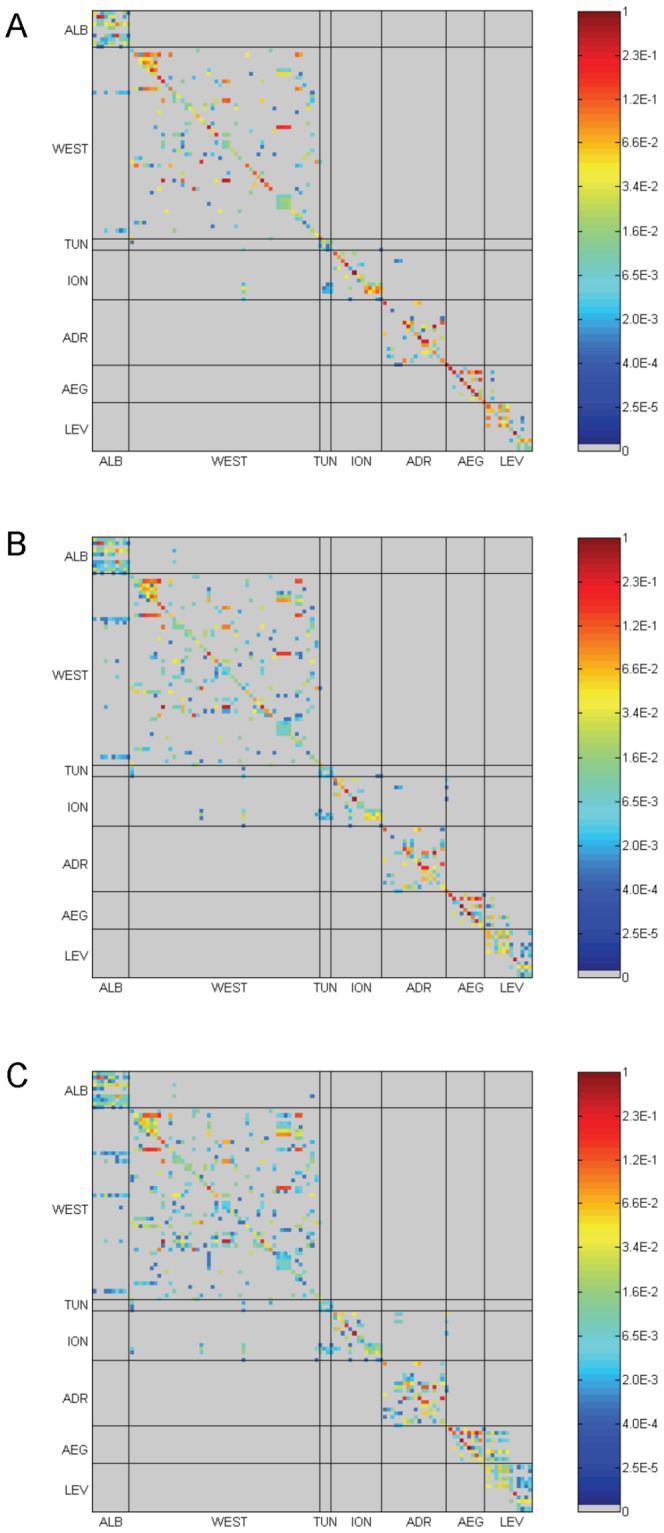
Connectivity matrices. Colors represent the probabilities that a larva born in MPA *j* (column) is transported to MPA *i* (line). MPAs are first sorted by ecoregion [108], then alphabetically within ecoregions. A, PLD = 20 days; B, PLD = 30 days; C, PLD = 40 days. ALB, Alboran Sea; WEST, Western Mediterranean; TUN, Tunisian Plateau and Gulf of Sidra; ION, Ionian Sea; ADR, Adriatic Sea; AEG, Aegean Sea; LEV, Levantine Sea.

**Figure 4:**
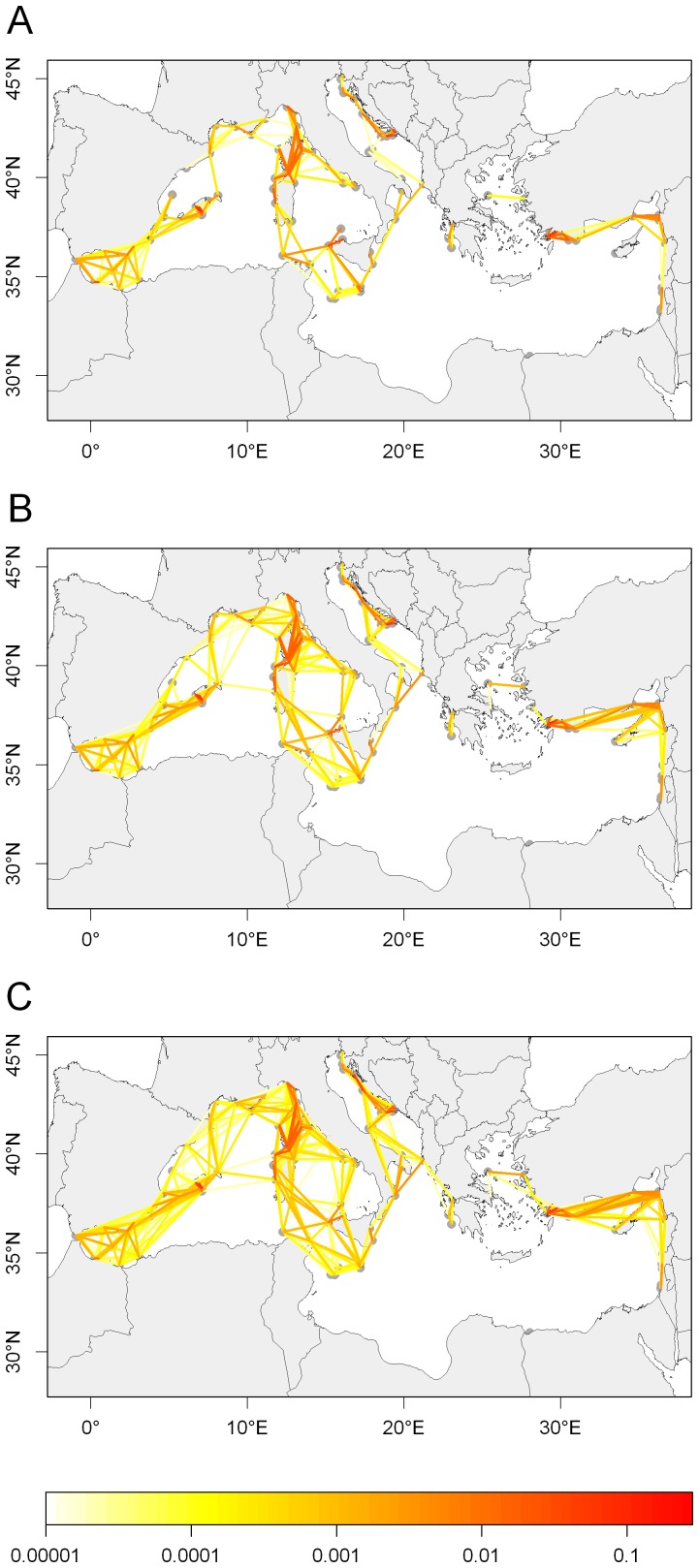
Connections between MPAs. Colors represent the connection probability. A, PLD = 20 days; B, PLD = 30 days; C, PLD = 40 days.

Out of 13,225 possible connections in the connectivity matrix, connectance was 0.0482 (Figure 3). When MPAs were connected, the connection probability was always very low (median 0.00028, interquartile range 0.0017) (Figure 4). Non-zero connection probabilities were all larger than 0.00001, but only 30.93% of non-zero connections (1.50% of the total number of possible connections) were larger than 0.001. Of these 30.93%, 9.11% were self-loops and 21.82% were between-MPAs connections.

### Neighborhood and Clusters

Neighborhood sizes were small (Figure S2–S3). Downstream neighborhood sizes were comprised between 0 and 12 (median 4, interquartile range 4) and upstream neighborhood sizes varied between 0 and 13 (median 4, interquartile range 4). Downstream and upstream neighborhood sizes were correlated (Spearman’s rank correlation *ρ* = 0.38, *p*<0.0001), meaning that MPAs contributing larvae downstream to many other MPAs are also receiving larvae by many upstream neighbors. However, for some MPAs, there were large differences between the number of incoming and outgoing connections (Figure S4). One MPA (Sallum, Egypt) was completely isolated (upstream and downstream neighborhood size = 0), 7 MPAs were pure sources (upstream neighbor size = 0) and 2 were pure sinks (downstream neighborhood size = 0).

**Figure 5:**
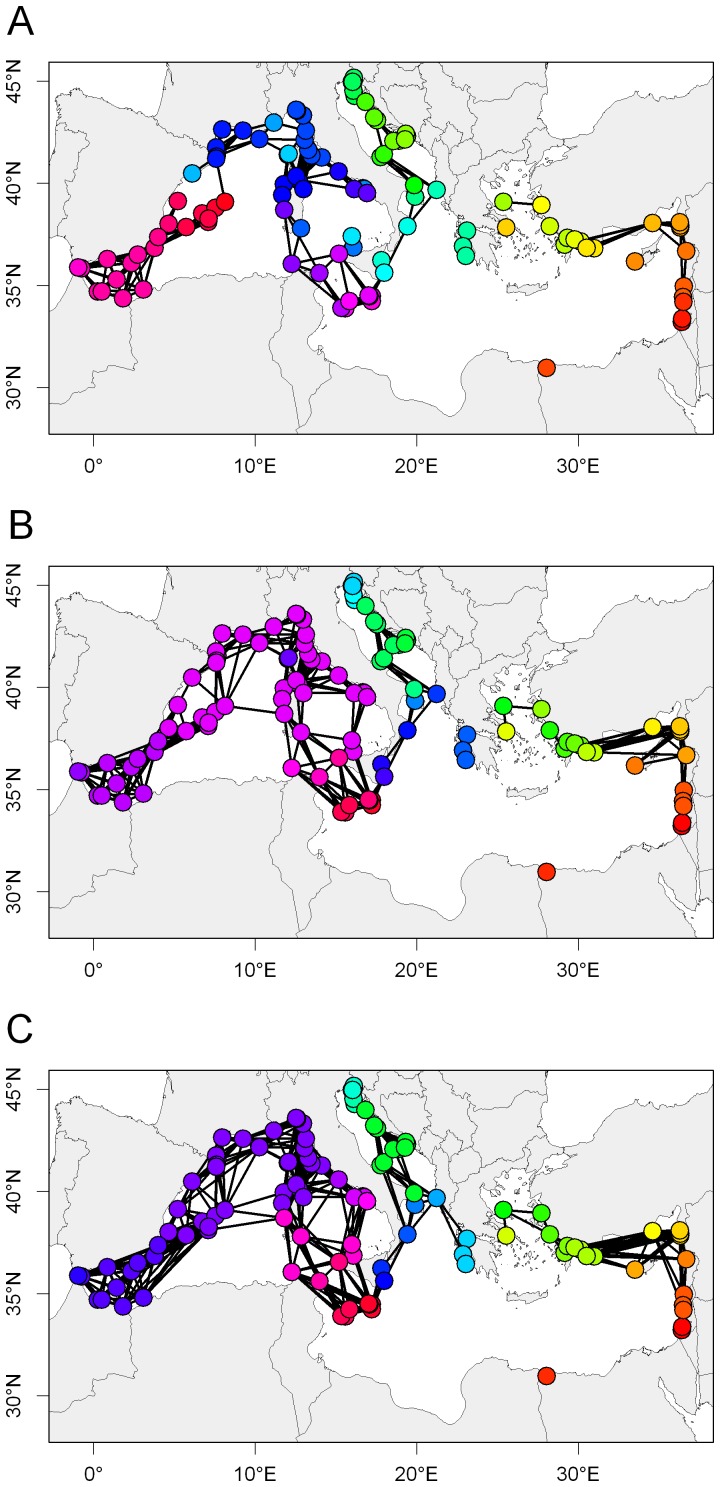
Clusters. Colors represent clusters, identified using a ‘strong’ connectivity criterion (see methods). A, PLD = 20 days; B, PLD = 30 days; C, PLD = 40 days.D.

MPAs were not all comprised in a single cluster (Figure 5). MPAs were grouped into 38 strongly connected clusters and 5 weakly connected clusters. Among the strongly connected clusters, the largest one was formed by 43 MPAs located in the Western Mediterranean ecoregion (referred to as the Western Mediterranean cluster in the following) and the second largest one was formed by 11 MPAs located in the Alboran and Western Mediterranean ecoregions (referred to as the Alboran cluster in the following). The other clusters were smaller and constituted by 1–7 MPAs. The large Western Mediterranean cluster was lost when PLD = 20 days, while the Alboran one persisted. Even if the number of clusters decreased as PLD and connectivity increased (compare the three panels of Figure 5), the system was never fully connected whatever the scenario. At the longest PLD (40 days), MPAs were grouped into 36 strongly connected clusters, the largest of which were the Western Mediterranean cluster (36 MPAs), the Alboran cluster (11) and a Southern Adriatic cluster (11).

The spawning month also had an effect on clustering (Figure S5). The large Western Mediterranean cluster was lost when spawning occurred in July and was smaller when spawning occurred in September. The Alboran cluster persisted whatever the spawning month.

### Larval Export

**Figure 6:**
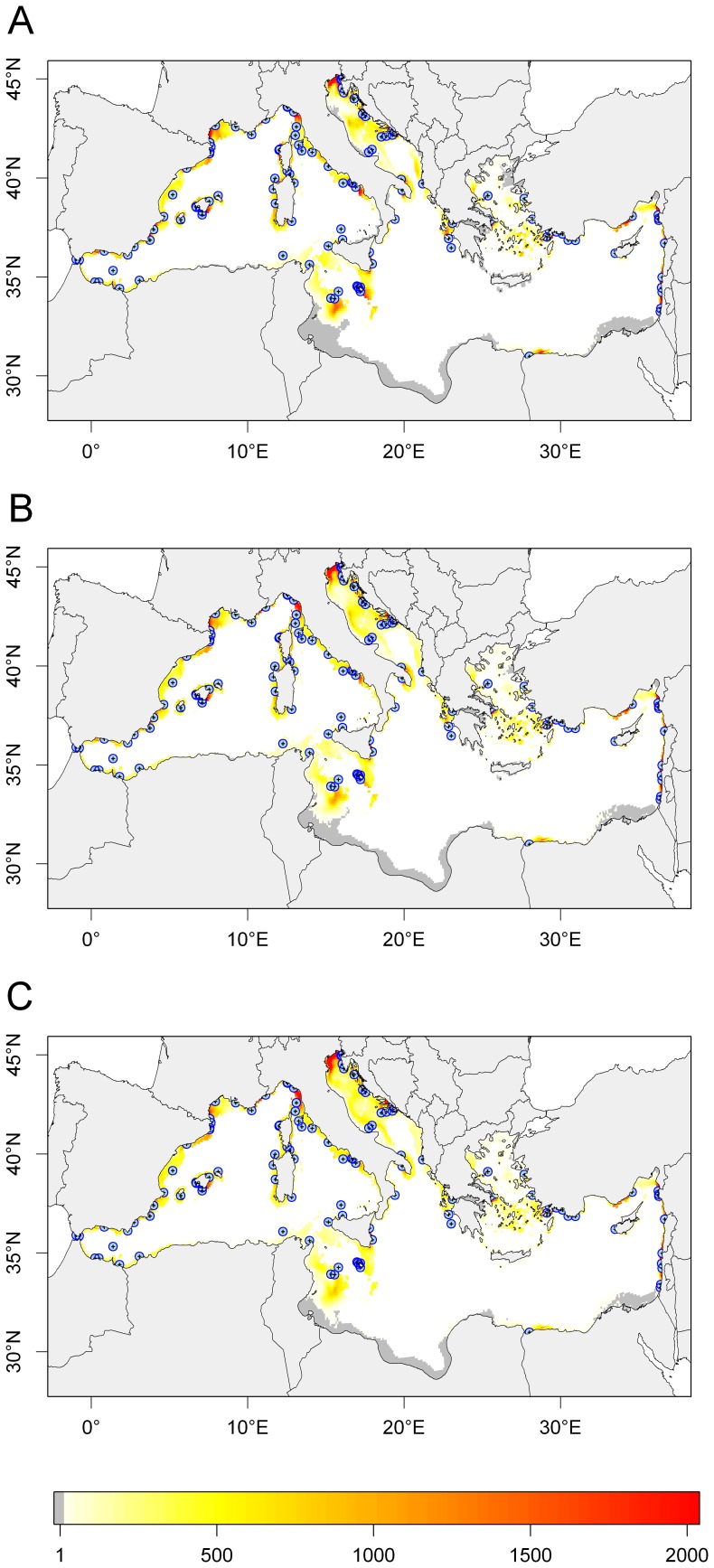
Larval abundance over coastal areas. Abundance of larvae on the continental shelf (<200 m depth) for all MPAs at the end of larval transport. A, PLD = 20 days; B, PLD = 30 days; C, PLD = 40 days.

At the end of larval transport, larvae were found in open sea waters (55.8%) or on the continental shelf (44.2%) (Figure 6). Only 2.1% of larvae were found within MPAs. There were vast areas of the continental shelf not receiving larvae from MPAs: on the Tunisian plateau, on the Libyan and Egyptian shelf and on the Aegean shelf. MPAs are absent in these regions, and larval dispersal distances are not large enough to reach them. The percentage of coastal areas that did not receive larvae from MPAs (grey in Figure 6) was 22.2%.

Even if the fraction of coastline receiving larvae from MPAs was high, the abundance of arriving larvae was low. Larval abundance decreased very steeply with distance from the release point (Figure S6), with 50% of larvae dispersing within 100 km from their release point. High larval abundances were found in the Balearic Islands, the Gulf of Lion, the Ligurian Sea, the Gulf of Trieste, the Northern Adriatic Sea, the Southern Turkish Coast (Göksu deltası) and the Israeli and Lebanese coasts. Larval abundance was not affected by the density of surrounding MPAs: there were areas with high density of MPAs and low larval abundance, *e.g.* Sardinia, and areas with high larval abundance around a single, isolated MPA (*e.g.* Göksu deltası). In the case of Sardinia, the narrowness of the continental shelf seems responsible for the low larval abundance, as the majority of larvae were lost in open sea waters.

The maps illustrating larval export (Figure 6) are based on the hypothesis that all MPAs produce the same number of larvae (50,000 in our case). We also drew maps under the hypothesis that larval production is proportional to MPA size (Figure S7). These maps showed that MPA larval production affects the spatial pattern of larval abundance. For example, high abundances were found in the Aegean Sea, as a consequence of the large surface area of the Alonissos-Vories Sporades MPA.

The spawning month had a slight effect on the spatial pattern of larval abundance (Figure S8). The areas not receiving larvae and the areas of high larval abundance did not change, but the concentration of larvae in areas of high abundance was stronger for July and weaker for September compared to August.

### Betweenness Centrality

**Figure 7:**
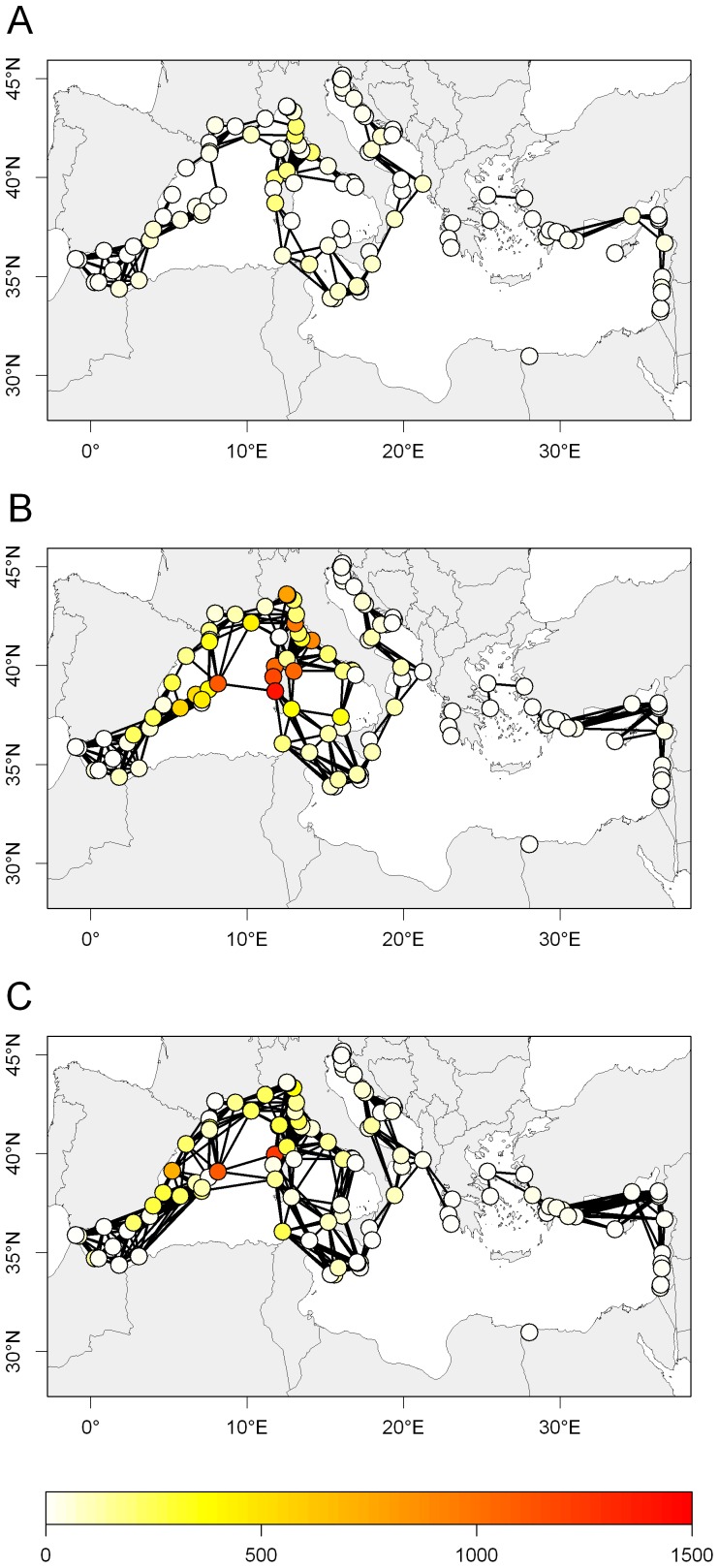
Betweenness centrality of MPAs. A, PLD = 20 days; B, PLD = 30 days; C, PLD = 40 days.

The betweenness centrality was very variable across MPAs and geographical locations (Figure 7). In the Western Mediterranean, betweenness centrality was generally higher than in the other ecoregions due to the central positions occupied by the MPAs in this ecoregion. In particular, the connection from Penisola del Sinis to Norte de Menorca conferred high betweenness centrality to these MPAs, which emerged thus as crucial nodes to ensure the connectivity between the Western Mediterranean basin and the Tyrrhenian basin. The importance of this connection emerged as it was lost (under short PLD) or accompanied by two more connections (under long PLD). The loss of this connection under short PLD (Figure 7A) changed dramatically the distribution of betweenness centralities, as the system became more fragmented (higher number of clusters and fewer paths). The emerging of more connections between the Balearic Islands and Sardinia with longer PLD (Figure 7C) lowered the betweenness centralities of Penisola del Sinis and Norte de Menorca.

The spawning month had also a great effect on betweenness centrality (Figure S9). July and September showed lower values of betweenness centralities, in particular for the two MPAs that exhibited higher values in August (Penisola del Sinis and Norte de Menorca), due to the loss of the connection between them.

### Effects of PLD, Vertical Migration and Spawning Month

The effects of PLD on dispersal distances, connectivity and larval export are further illustrated in Table 1 and Figure S10. Larval dispersal distances increased linearly with PLD. Connectivity increased with PLD as shown by the increase in connectance and the decrease in the number of clusters. Larval export to fished areas decreased with PLD: the fraction of larvae retained on the continental shelf decreased, meaning that a greater fraction of larvae was lost in open sea waters. The fraction of shelf area not receiving larvae from MPAs decreased also, as a result of increased advection and longer dispersal distances. The decrease in larval retention fraction and increase in the areas of the continental shelf receiving larvae means that the overall density of larvae on the continental shelf is lower as PLD increases.

**Table 1:** Effect of PLD on dispersal distances, connectivity and larval export.

Variable	Slope	adj-*R^2^*	*p*
Dispersal distance (median)	3.12±0.02	0.999	<2.2⋅10^−16^
Dispersal distance (75^th^ percentile)	5.16±0.07	0.996	<2.2⋅10^−16^
Connectance	0.001174±0.000022	0.992	<2.2⋅10^−16^
Number of strongly connected clusters	−1.24±0.12	0.831	5.57⋅10^−9^
Number of weakly connected clusters	−0.28±0.028	0.833	4.89⋅10^−9^
Fraction of larvae retained on continental shelf	−0.00366±0.00009	0.989	<2.2⋅10^−16^
Fraction of shelf area not receiving larvae	−0.00596±0.00018	0.981	<2.2⋅10^−16^

The effect of PLD on the variables was tested using linear regression. The slope of the regression lines is the increase in the response variable for a one-day increase in PLD.

Vertical migration decreased dispersal (Table 2). Larval dispersal distances were smaller and connectance was lower in the vertical migration scenario than in the passive dispersal scenario. Vertical migration increased the number of clusters, larval retention on the continental shelf and the fraction of shelf area that did not receive larvae from MPAs. Thus, the effects of vertical migration were comparable to those of reducing PLD. Approximately, the vertical migration behavior simulated here (from 0.2 m 50 m deep at 8am and from 50 m to 0.2 m deep at 8 pm) had the same effect as a reduction of PLD from 30 days to 20–22 days.

**Table 2:** Effect of vertical migration on dispersal distances, connectivity and larval export.

Variable	Without vertical migration	With vertical migration	*p*
Dispersal distance (median)	102.89±0.03	73.66±0.04	1.49E−49
Dispersal distance (75^th^ percentile)	196.67±0.05	155.02±0.10	1.98E−45
Connectance	0.0476±0.0005	0.0371±0.0005	3.54E−20
Number of strongly connected clusters	46.6±3.5	61.3±1.6	4.33E−10
Number of weakly connected clusters	6.1±0.7	10.6±1.0	7.52E−10
Fraction of larvae retained on continental shelf	0.4414±0.0002	0.4827±0.0002	8.03E−39
Fraction of shelf area not receiving larvae	0.2231±0.0014	0.2997±0.0011	1.70E−28

Values are mean ± standard deviation on 10 replicates. Differences in variables between scenarios with and without vertical migration were tested using Student’s *t* test.

The spawning month (July, August or September) had a significant effect on dispersal (Table 3). Dispersal distance decreased from July to September, as a result of the weakening of coastal current velocity (i.e. mean velocity July>mean velocity August>mean velocity September). The origin of this weakening is however not clear: wind and Atlantic water input did not show a similar weakening pattern over time. The reduction in current velocity resulted also in a smaller number of clusters and increased the fraction of shelf area that did not receive larvae.

**Table 3:** Effect of spawning season on dispersal distances, connectivity and larval export.

Variable	July	August	September	*p*
Dispersal distance (median)	112.98±0.04	102.89±0.03	78.93±0.03	2.00E−72
Dispersal distance (75^th^ percentile)	202.55±0.08	196.67±0.05	165.93±0.06	3.99E−66
Connectance	0.0420±0.0004	0.0476±0.0005	0.0449±0.0004	1.40E−19
Number of strongly connected clusters	66.1±2.0	46.6±3.5	40.9±2.6	1.34E−17
Number of weakly connected clusters	6.1±0.6	6.1±0.7	6.4±1.2	0.67
Fraction of larvae retained on continental shelf	0.4479±0.0002	0.4414±0.0002	0.4695±0.0002	6.14E−49
Fraction of shelf area not receiving larvae	0.2207±0.0015	0.2231±0.0014	0.2538±0.0016	1.92E−28

Values are mean ± standard deviation on 10 replicates. Differences in variables between spawning seasons were tested using ANOVA.

## Discussion

Throughout the world, MPAs are increasingly considered to be effective tools for protecting biodiversity and sustaining fisheries, but their success depends on connectivity and larval dispersal [7], [9]–[13]. Taking *E. marginatus* as a case study, we have shown that *i*) the system of Mediterranean MPAs is not a fully connected network and is composed of several independent clusters, *ii*) larval export to the continental shelf is limited with about 20% of coastal areas not receiving larvae and *iii*) single MPAs (nodes) may play a very important role in assuring the connectivity of the whole system (high betweenness centrality).

### Model Strengths and Limitations

Previous studies on MPAs employed genetic or microchemical methods [10], [16]–[23] to infer patterns of connectivity and larval export. However, these techniques are adequate for studies at small scales and for small networks, but hardly applicable at larger scales, given the high costs and intensive sampling efforts required for the analyses. Ideally, combining genetic, microchemical and biophysical modeling methods should be used together to overcome the strengths and weaknesses of each [8]. This study is the first assessment of connectivity between numerous MPAs at the scale of an entire Sea. The Mediterranean is currently protected by a system of around a hundred MPAs with varying degrees of protection. Recent analyses have identified critical factors controlling the effectiveness of MPAs, such as the size and the age since implementation [4], [6], and urged for the evaluation of connectivity of the entire system. Our biophysical model can be considered a first step towards the accomplishment of this task.

Nonetheless, our analyses suffer from four types of limitations. First, although the horizontal grid size of NEMOMED12 (1/12° of degree, between 6 and 8 km, from North to South) is one of the smallest available for modeling the entire Mediterranean basin [51], [80], larval retention within coastal waters and connectivity may be influenced by hydrodynamic processes acting at even smaller scales [70]. Topographical features such as headlands and semi-enclosed bays can generate eddies capable of retaining larvae near the coast [54]. Indeed, larval dispersal toward fished areas can be limited in space and smaller scale studies using higher resolution models may help describe the patterns of demographically relevant larval export [81], [82].

Secondly, directional active swimming, orientation mechanisms, and interaction with resident fishes can change the patterns of larval dispersal predicted under the assumption of passive transport and increase the chances of retention and recruitment [83]–[85]. Behavioral studies on dusky grouper larvae are lacking, but would be beneficial for understanding these biological processes [83].

Thirdly, the assumption that all MPAs are effectively enforcing fishing restrictions is likely unrealistic, which can introduce an upward bias in estimates of larval production, connectivity and larval export. Finally, spawning of groupers can occur also in unprotected areas: connections between MPAs and larval abundances could be strengthened by larvae spawned outside MPAs and be higher than those estimated here.

In light of these limitations, the results of our study must be interpreted with caution. The analyses could be improved with the availability of models with finer spatial resolution, better knowledge on grouper adult and larval biology and extensive data on the effects of MPAs on fertility, survival and biomass of groupers. As an alternative, modeling studies on connectivity between MPAs may be conducted at smaller spatial scales, for which models with better resolution exist [81], and using other species with different dispersal strategy.

We have conducted an explicit sensitivity analysis on the effects of pelagic larval duration, vertical migration and spawning month on the model results. The uncertainty surrounding larval and adult biology resulted in considerable variation in connectivity and larval export estimates. Moreover, it showed that vertical migration and PLD are equally important sources of variation on all measures of larval dispersal, connectivity and larval export. Despite all these limitations, our study remains highly conservative by considering an optimistic scenario of connectivity in the Mediterranean Sea (species with long pelagic larval duration and all MPAs effectively enforcing fishing restrictions) while accounting for uncertainty.

### Low Connectivity between Mediterranean MPAs

The main result of our work is that Mediterranean MPAs does not form a well-connected network. We calculated the first-order neighborhood sizes of MPAs and found that over a single reproductive season, larvae can spread to a limited number of neighbors. We also identified clusters to picture the potential for multigenerational connectivity beyond first-order neighborhoods. The number of identified clusters was high, highlighting the weak connectivity of Mediterranean MPAs. Clusters must be interpreted bearing in mind the timescales of multigenerational connectivity and MPA implementation. The generation time of the dusky grouper is likely of the order of several years, because sexually mature individuals are observed after age 6 [36]. MPA implementation follows the recommendation of national and international policy. In 2010, the Convention on Biological Diversity (CBD) set specific targets to implement new MPAs by 2020 (the ‘Aichi targets’; www.cbd.int/cop10). As the system of Mediterranean MPAs will be integrated by new MPAs in the following 5–10 years, connectivity will be strengthened and some clusters will merge. In this perspective, combining our analyses with an algorithm of reserve design may help to find the optimal configuration of MPAs for which connectivity is maximized [86]–[89].

We used a strong connectivity criterion to identify clusters, which implies the possibility of reaching every node of the cluster starting from any node. With this criterion, MPAs lacking incoming connections cannot be included in a cluster with other MPAs. Similarly, MPAs lacking outgoing connections are not included in a cluster with other MPAs. In other words, each pure source and each pure sink defines a single cluster. In clusters formed by more than one MPA, all MPAs act as both sources and sinks. The ‘weak’ connectivity criterion is less stringent and, not surprisingly, it led to the identification of fewer clusters (Figure S11). Weakly connected clusters can be misleading, because they incorrectly picture sources as reachable locations from other MPAs, and sinks as potential sources of larvae for other nodes. Source-sink dynamics is correctly accounted for only using a strong connectivity criterion, and can have important implications for population dynamics and genetic differentiation [41].

In addition to the small number of connections per MPA (small neighborhood size) and the small number of paths connecting distant MPAs (high number of clusters), we also observed that connection probabilities were always small. The connection probability determines whether larval dispersal affects demography and population genetics through its effect on the migration rates between populations. Low migration rates are sufficient for erasing genetic differentiation but not for creating demographic links between populations [90]. Treml et al. [91] used a connectivity threshold of 0.001, above which connection probabilities were considered demographically relevant. In our case, only one third of the connection probabilities between Mediterranean MPAs were above this value. The effects of these connections on population persistence can be assessed by using one of the numerous models available to study population dynamics and persistence in subdivided populations [92], [93] or specifically for MPAs [74], [94]. Connections with probability below 0.001 are likely insufficient to affect the demography of recipient populations, but can be considered high enough for genetic differentiation and evolution.

The identified connections among MPAs followed known patterns of sea water circulation in the Mediterranean Sea [64]. In the Alboran Sea, Atlantic water produces persistent clockwise gyres that favor connectivity among MPAs, which showed frequent bidirectional connections (see the upper-left quadrant of the connectivity matrix, Figure 3). In the Western Mediterranean, currents flow from West to East along the southern coast, but we did not observe connections between the Alboran and the Tunisian MPAs, because they are separated by a too large distance. Conversely, in the North-Western Mediterranean Sea, currents flow from East to West (Northern Current) and the higher density of MPAs on Italian, French and Spanish coasts ensures sufficient connectivity in this zone. The Balearic Islands could act as a bridge between the Southern and the Northern coasts thanks to the formation of anticyclonic eddies North of Algeria if there were MPAs acting as sources of larvae on the central coast of Algeria. In the Tyrrhenian Sea, although the main currents flow from South to North along the Italian coast, we observed directional connectivity from Northern to Southern MPAs; this is linked to a complex circulation in the interior of this sea, with both cyclonic and anticyclonic eddies. In the Adriatic Sea, connections were often bidirectional following the main cyclonic circulation, flowing northward along the Illyrian coast and southward along the Italian coast. Conversely, connections in the Ionian Sea were mostly unidirectional (Figure 3), from Apulia to Malta, consistently with the anticyclonic surface circulation occurring in the northern Ionian Sea during the mid 2000s. MPAs of Israel, Lebanon, Syria and Turkey form a well connected cluster thanks to the coastal surface current (Libyo-Egyptian Current and then Asia Minor Current) flowing counterclockwise from Egypt towards Turkey and Greece.

Our results apply only to the dusky grouper in the system of Mediterranean MPAs. Other Mediterranean fishes will likely show lower levels of connectivity, as connectivity increases with PLD and the dusky grouper has one of the longest PLD [95]. In other management systems, connectivity will depend on the number and spatial arrangement of MPAs. The Mediterranean Sea has many MPAs, which facilitate direct and multi-step connections. The effect of MPA density on connectivity is demonstrated by the higher connectivity of Western Mediterranean basin compared to the Eastern basin. In other areas of the globe, where MPAs are less numerous, connectivity will be likely lower than in the Mediterranean.

### Implications for Population Viability

Some MPAs did not receive larvae from other MPAs and recruitment was constituted only of locally produced larvae (i.e. a self-recruitment fraction of 1). This corroborates the results of microchemical analyses of larval otolith [10], [18] and genetic parentage analyses [16], [19], which suggest that local retention is much higher than previously thought and makes important contribution to recruitment. High levels of self-recruitment and low connectivity are also observed in other models of larval dispersal [84], [85]. These models integrate a simulation of active swimming and physiological sensory mechanisms, features that increase larval retention. In our case, high self-recruitment fractions are found using passive dispersal only.

Low connectivity and high self-recruitment can have deleterious consequences for the adaptation of local populations. Although populations relying on self-recruitment may be demographically viable if the number of recruits is large enough to replace dead individuals, lack of larval supply from other populations can lead to inbreeding depression. This happens when closely related recruits grow and interbreed, producing inbred offspring, after which inbreeding depression can increase the risk of population extinction [96]. In addition, isolated populations are not reachable by novel, adaptive alleles emerging in other populations by mutation. The lack of connectivity may thus reduce the possibility to adapt to changing environmental conditions.

This is relevant in the context of global climate change [97], [98]. Sea surface temperature is expected to increase by about 3°C in the Mediterranean Sea by the end of the 21^st^ century [99], [100]. Warming temperatures may displace the climatic niches of many Mediterranean fishes and lead one-fifth of endemic Mediterranean fishes to extinction [101], [102]. Although the dusky grouper is present outside the Mediterranean Sea and is thought to fare well in warm waters [103], locally adapted populations in the Northern Mediterranean may not be sufficiently adapted to warm temperatures. A lack of gene flow from Southern, warm-adapted populations may impair the persistence of the species in the Northern Mediterranean.

Population connectivity between the Northern and Southern coasts of the Mediterranean Sea has been the object of several population genetics studies. Although analyses on *E. marginatus* revealed strong connections between the Northern and the Southern coasts of the Western Mediterranean basin, they also identified barriers to dispersal associated with oceanographic dynamics and revealed that the species is not panmictic [104], [105]. As for the Western Mediterranean basin, the genetic similarity between populations inhabiting the Northern and Southern coasts may reflect historical rather than contemporary gene flow and may not be indicative of present-day connectivity [106]. Moreover, past population genetics analyses did not include samples from the Eastern Mediterranean basin (apart for one sample from Greece, [104]) and the existence of connections between Southern and Northern Mediterranean coasts is still disputable.

It is important that future studies investigate adaptive genetic variation and identify regions of the genome that are under selection, as a first step to describe the geographic distribution of adaptive genetic variation and to understand local adaptation. Populations that harbor unique genetic variants should be prioritized for conservation. In particular, genetic variants conferring the ability to resist thermal stress may be fundamental to allow the species to adapt to climate change. These prioritized populations should be preferentially located in zones that can act as sources for more vulnerable populations [98].

As discussed above, it should not be forgotten that first order connections allow for dispersal to few neighboring populations, while multigenerational connectivity is impaired by the long generation time of the dusky grouper. Thus, gene flow across the network likely occurs over decades or hundreds of years, potentially limiting the spread of adaptive novel mutations and adaptation to climate change.

### Larval Export

Larval export from MPAs to fished areas resulted in great variability in larval abundances on the continental shelf at the end of larval transport. In the Southern Mediterranean, vast areas of the continental shelf are not expected to receive any larvae at all from MPAs (gray areas in Figure 6), as a result of their large distance from MPAs and low dispersal. Conversely, the occurrence of high abundances (red in Figure 6) was more complex to explain. Areas with high MPA density were not consistently associated with high larval abundances, therefore showing that a high density of MPAs is not sufficient to ensure high larval export to fished areas. The direction of sea currents and the surface area of the continental shelf seem to better explain the spatial patterns of larval export. Where the continental shelf is large, larvae can accumulate even if they disperse far away from their release point. The direction of sea currents is also important, as currents can transport larvae far away offshore in regions not suitable for settlement. In our model, habitat was not explicitly modeled, so the illustrated patterns should be interpreted as potential settlement conditional of habitat availability (i.e. rocky shores and rocky bottom for the dusky grouper).

The potential for larval supply to fished areas beyond MPA borders remains still an open question, as recruitment subsidy is dependent on the differential in productivity between MPAs and fished areas [107]. Without data on larval production within and outside MPA boundaries, it remains challenging to predict whether the amount of exported larvae is sufficient to create recruitment supply. Moreover, crucial knowledge on pelagic larval mortality and post-settlement mortality is lacking to correctly assess recruitment benefits in relation to the increase in spawning stock biomass within reserves.

### Betweenness Centrality: Importance of Individual MPAs

Beyond first-order connections, connectivity among MPAs was possible through multi-step connections, resulting in groups (clusters) of MPAs among which connectivity was assured in a strong or a weak sense. Multi-step connections take advantage of single MPAs acting as central nodes to spread genes between nodes that are not directly connected. The betweenness centrality measures the importance of such central MPAs for multi-step, multi-generational gene transfers [76].

The Penisola del Sinis (in Sardinia) and Norte de Menorca (in the Balearic Islands) show particularly high betweenness centralities, meaning they are obligate gateways for the passage of genes from the Western Mediterranean towards the Tyrrhenian basin. The high number of MPAs in the North-Western Mediterranean and the central position of the Balearic Islands and Sardinia increase the chance of having high betweenness centrality for the MPAs located on these islands. Since Mediterranean MPAs are exclusively on coasts or around islands, islands can act as natural bridges connecting distant regions. However, this does not mean that islands will always be central in the connectivity network. For example, the Pelagie islands, which lie at the geographic centre of the Mediterranean Sea on the Tunisian plateau, did not exhibit high betweenness centralities. This was due to the low number of MPAs in the Southeastern Mediterranean and notably to the absence of MPAs on the Libyan coasts. The upcoming implementation of coastal protected areas in Libya will facilitate the connections between the Western and the Eastern basins and may result in higher betweenness centrality for the Pelagie islands.

The four MPAs having the highest betweenness centralities (Penisola del Sinis, Asinara, Norte de Menorca and Capo Caccia - Isola Piana) owed their status to the existence of a single connection (edge) from Penisola del Sinis (in Sardinia) to Norte de Menorca (in the Balearic Islands). The other Sardinian MPAs were not connected to Norte de Menorca, suggesting that connections are highly dependent on the precise location of MPAs. Thus, a high density of protected areas separated by short distances does not necessarily imply high connectivity and placing MPAs on islands does not necessarily confer them a crucial role. The creation of new protected areas to facilitate and increase connectivity should therefore be based on careful planning and integrate explicit evaluations of connectivity patterns.

The high betweenness centrality of some MPAs confer them a crucial role for sustaining the connectivity of the whole network. This role should be interpreted bearing in mind the timescales of MPA implementation and multigenerational transfer. As more MPAs will be created, the clustering of MPAs will change, as already discussed above, and the increasing number of connections will reduce the importance of single links and single MPAs for assuring multi-generational connectivity.

### Conclusions

We used a biophysical model to evaluate self-recruitment, larval supply and connectivity for Mediterranean MPAs. On one hand, an evaluation of recruitment levels in Mediterranean MPAs and recruitment supply beyond MPA borders is challenging since crucial knowledge on larval biology and MPA productivity is still lacking. On the other hand, our study, despite being conservative and based on an optimistic scenario, shows potentially high levels of self-recruitment and low connectivity in the present system of MPAs, demonstrating their poor ability to work as a proper network, with potentially deleterious consequences for the persistence of the exploited populations.

The low level of connectivity evidenced for the current system of MPAs poses new challenges for the design of a future Mediterranean MPA network according to the Strategic Plan for Biodiversity adopted by the Convention on Biological Diversity (COP10; www.cbd.int/cop10), which requires at least 10% of coastal and marine areas protected by 2020. This network will need to be a functioning network that ensures connectivity within and among MPAs, in order to supply larvae all over the Mediterranean continental shelf. Its optimal design will also need to account for the economic and social costs deriving from restrictions on fishing activities. Finally, the connectivity patterns estimated here are likely to change as a result of the effects of global climate change on larval biology and species range. Design of future MPA networks cannot ignore these effects and further studies are needed to understand how larval connectivity will change and how these changes will affect the persistence of exploited species within and outside MPAs.

## Supporting Information

Figure S1 **Self recruitment.** For each MPA, self-recruitment and subsidy recruitment are plotted in percent of total recruitment.
(TIF)

Figure S2 **Neighborhood sizes.** Number of downstream neighbors for each MPA. A, PLD = 20 days; B, PLD = 30 days; C, PLD = 40 days.
(TIFF)

Figure S3 **Neighborhood sizes.** Number upstream neighbors for each MPA. A, PLD = 20 days; B, PLD = 30 days; C, PLD = 40 days.
(TIFF)

Figure S4 **Neighborhood sizes.** Difference (downstream neighborhood size - upstream neighborhood size, i.e. positive values mean that outgoing connections are more numerous than incoming connections) for each MPA. A, PLD = 20 days; B, PLD = 30 days; C, PLD = 40 days.
(TIFF)

Figure S5 **Effect of spawning month on clusters.** Colors represent clusters, identified using a ‘strong’ connectivity criterion (see methods).
(TIFF)

Figure S6 **Larval dispersal distances.** Frequency distribution of individual larval dispersal distances from their release point over all MPAs and years (*n* = 5750000).
(TIFF)

Figure S7 **Effect of MPA larval production on larval abundance.** Abundance of larvae on the continental shelf (<200 m depth) for all MPAs at the end of larval transport. MPA larval production was proportional to MPA size. A, PLD = 20 days; B, PLD = 30 days; C, PLD = 40 days.
(TIFF)

Figure S8 **Effect of spawning month on larval abundance.** Abundance of larvae on the continental shelf (<200 m depth) for all MPAs at the end of larval transport.
(TIFF)

Figure S9 **Effect of spawning month on betweenness centrality.**
(TIFF)

Figure S10 **Effect of PLD on dispersal distances, connectivity and larval export.** Linear regression of dispersal distance on PLD (A, median dispersal distance; B, 75^th^ percentile of dispersal distance distribution), connectance on PLD (C), number of strongly connected clusters on PLD (D), fraction of larvae retained on the continental shelf on PLD (E) and fraction of shelf area not receiving larvae from MPA on PLD (F). Points are observed values and lines are estimated regression lines.
(EPS)

Figure S11 **Clusters (weak connectivity).** Colors represent clusters, identified using a ‘weak’ connectivity criterion (see methods). A, PLD = 20 days; B, PLD = 30 days; C, PLD = 40 days.
(TIFF)

Table S1 (XLS)
