# Supplementary figures and images for: Low Connectivity between Mediterranean Marine Protected Areas: A Biophysical Modeling Approach for the Dusky Grouper Epinephelus marginatus
Source: PLoS One. 2013 Jul 8;8(7):e68564. doi: 10.1371/journal.pone.0068564 (PMC3704643; doi:10.1371/journal.pone.0068564)

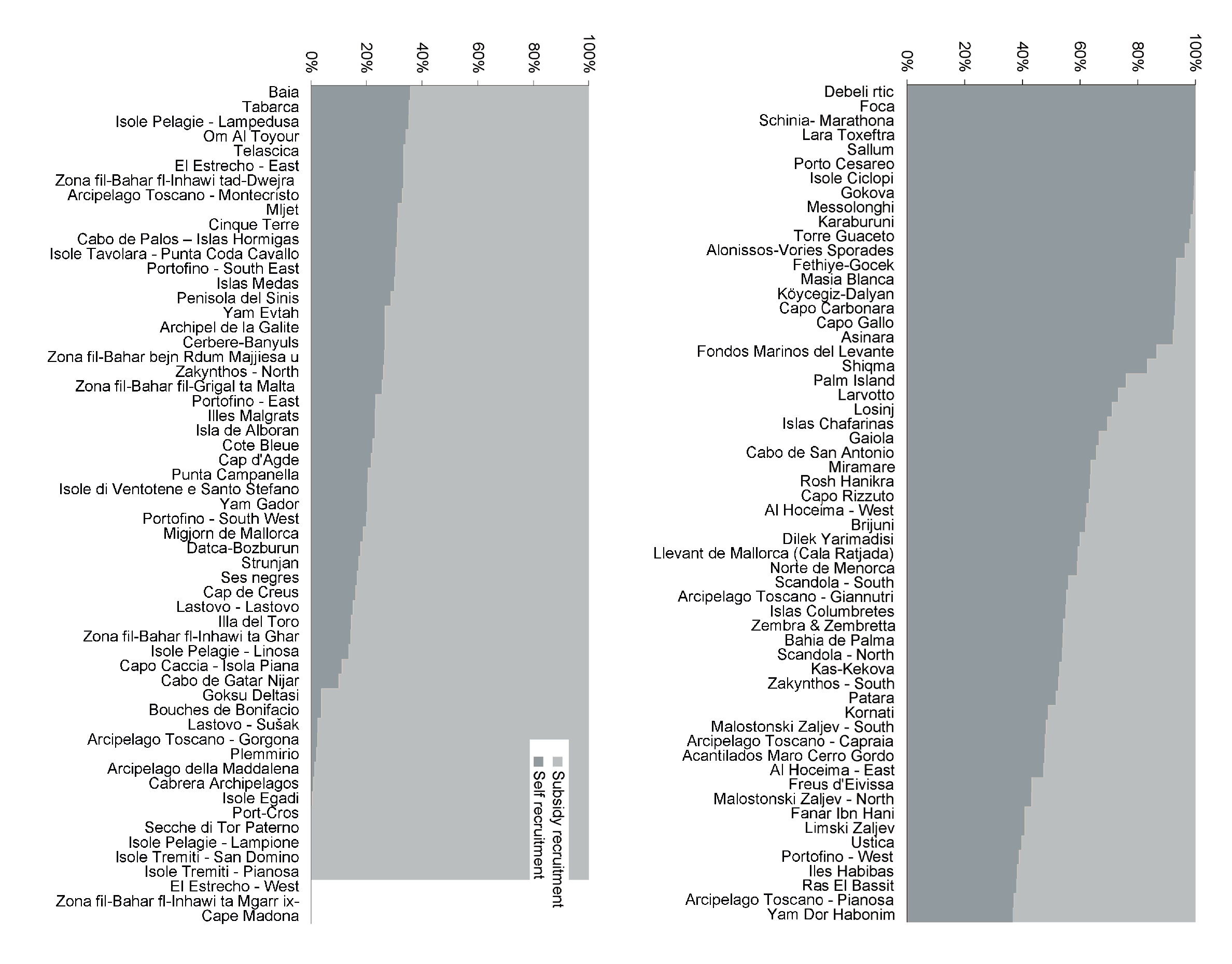

Supplement: Figure S1 — Self recruitment. For each MPA, self-recruitment and subsidy recruitment are plotted in percent of total recruitment. (TIF) [file pone.0068564.s001.tif]

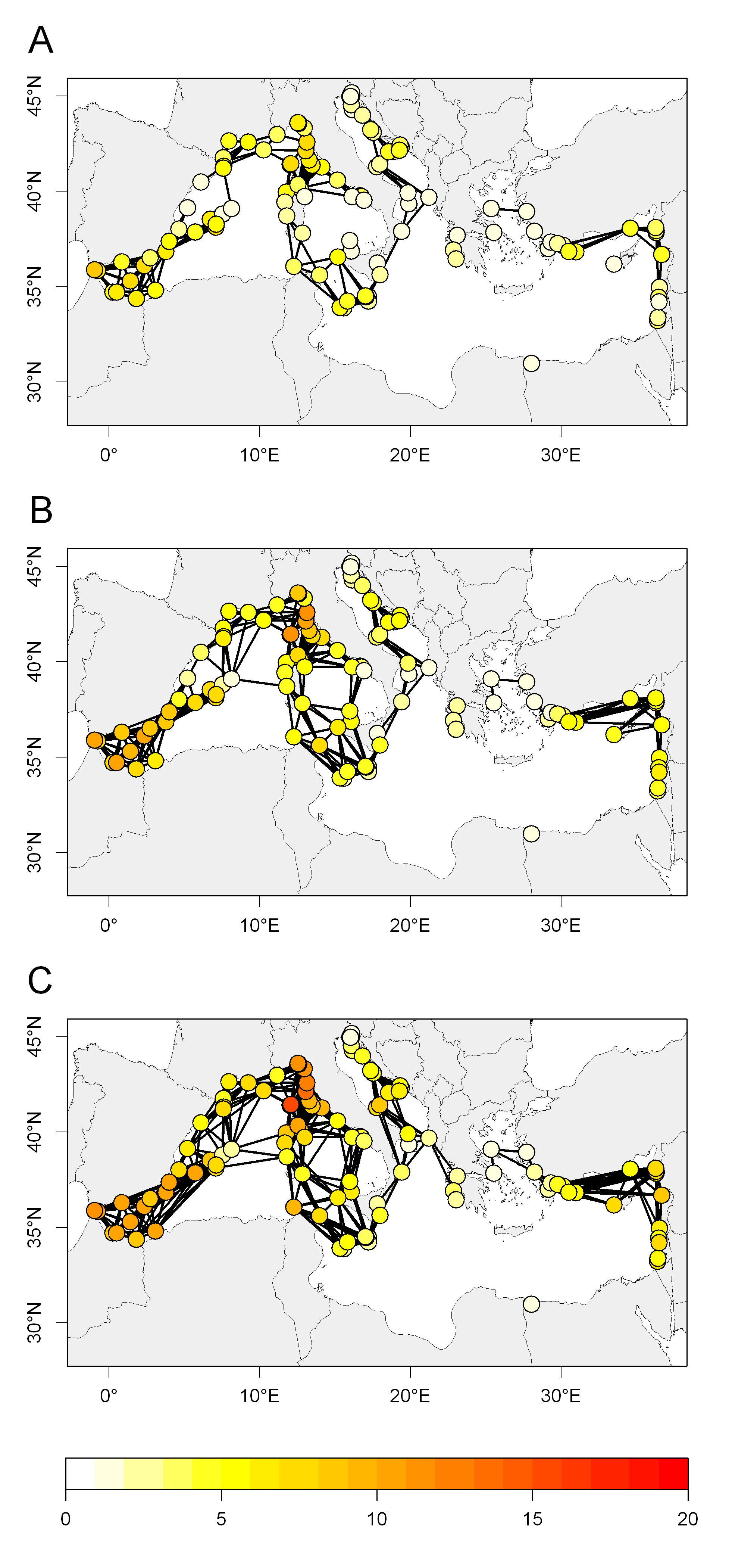

Supplement: Figure S2 — Neighborhood sizes. Number of downstream neighbors for each MPA. A, PLD = 20 days; B, PLD = 30 days; C, PLD = 40 days. (TIFF) [file pone.0068564.s002.tiff]

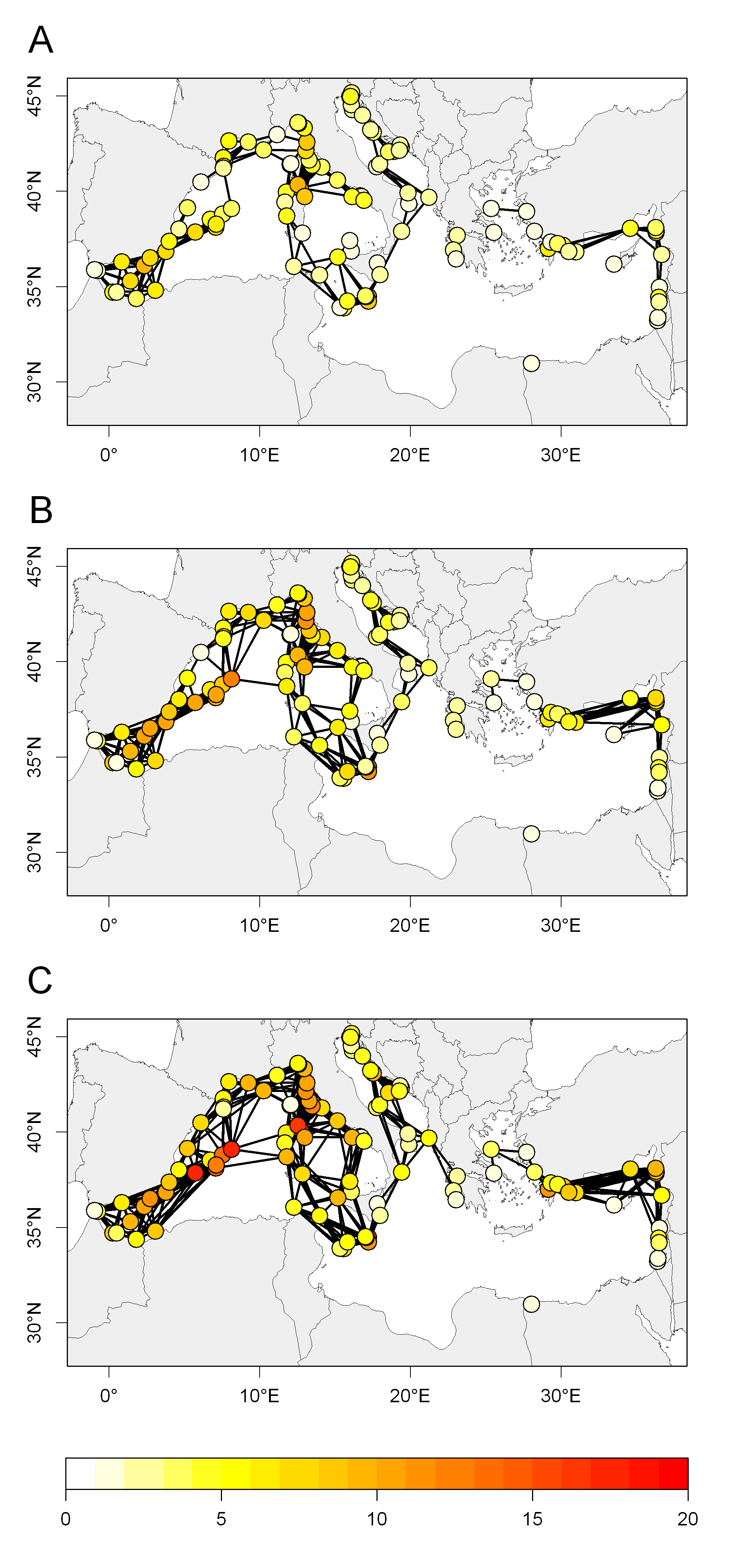

Supplement: Figure S3 — Neighborhood sizes. Number upstream neighbors for each MPA. A, PLD = 20 days; B, PLD = 30 days; C, PLD = 40 days. (TIFF) [file pone.0068564.s003.tiff]

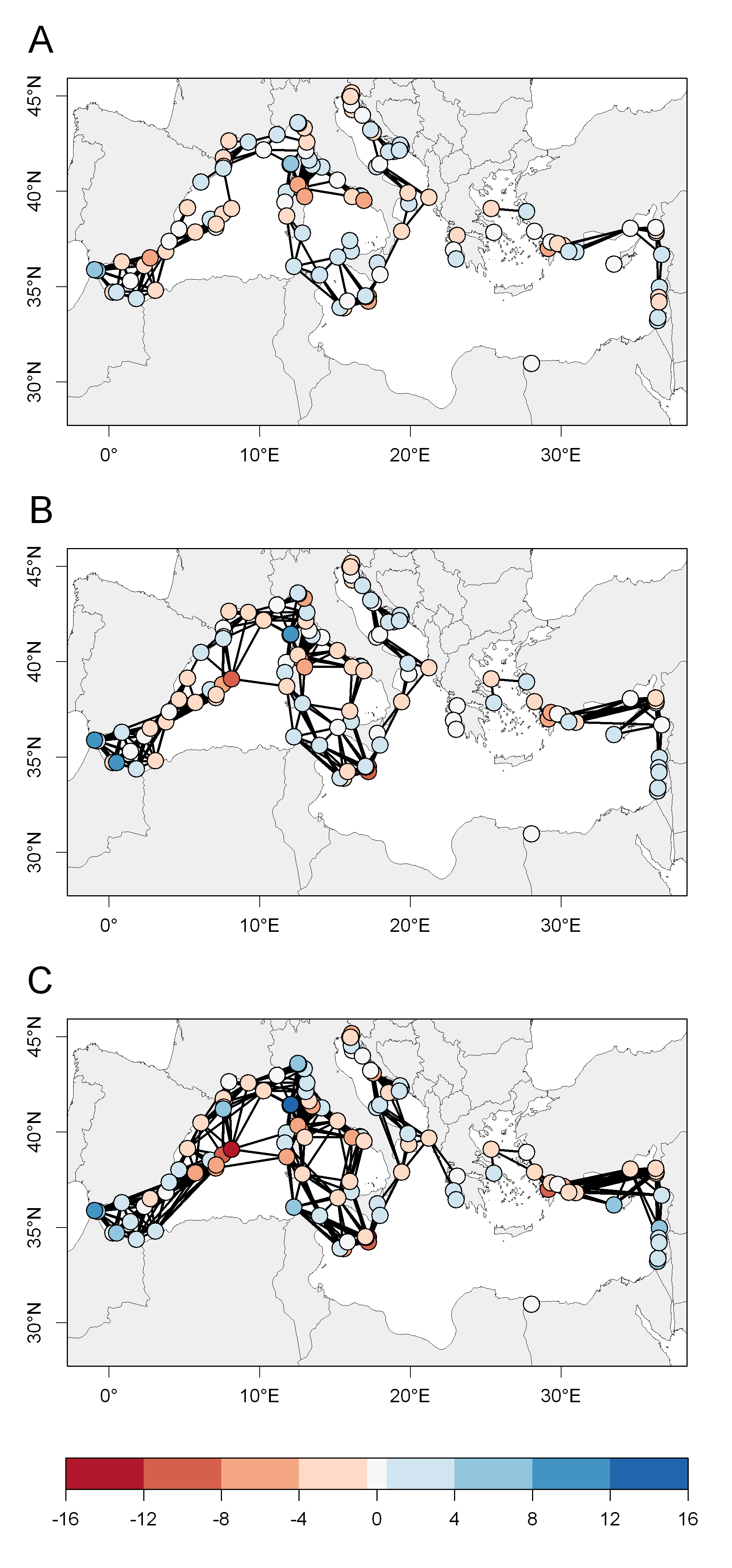

Supplement: Figure S4 — Neighborhood sizes. Difference (downstream neighborhood size - upstream neighborhood size, i.e. positive values mean that outgoing connections are more numerous than incoming connections) for each MPA. A, PLD = 20 days; B, PLD = 30 days; C, PLD = 40 days. (TIFF) [file pone.0068564.s004.tiff]

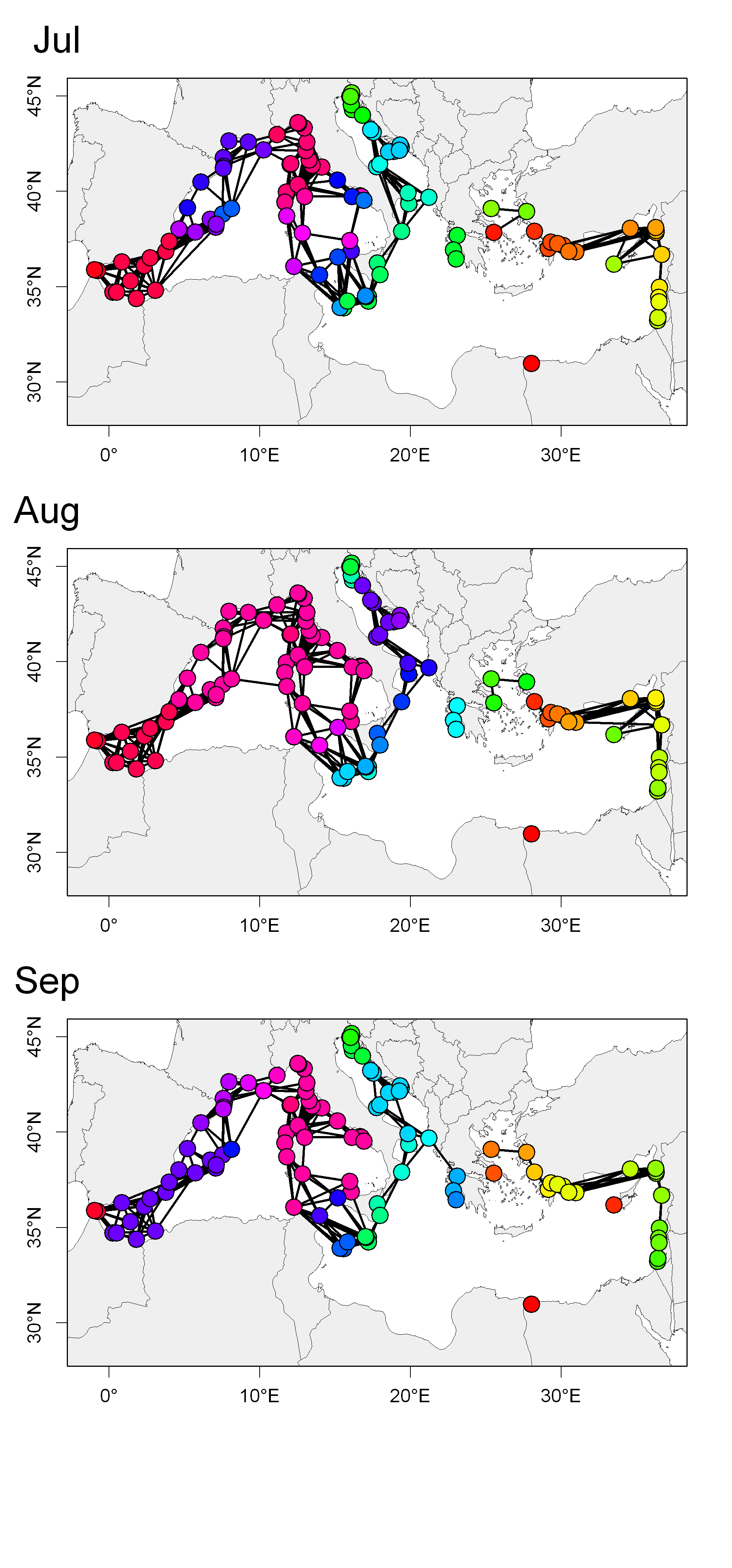

Supplement: Figure S5 — Effect of spawning month on clusters. Colors represent clusters, identified using a ‘strong’ connectivity criterion (see methods). (TIFF) [file pone.0068564.s005.tiff]

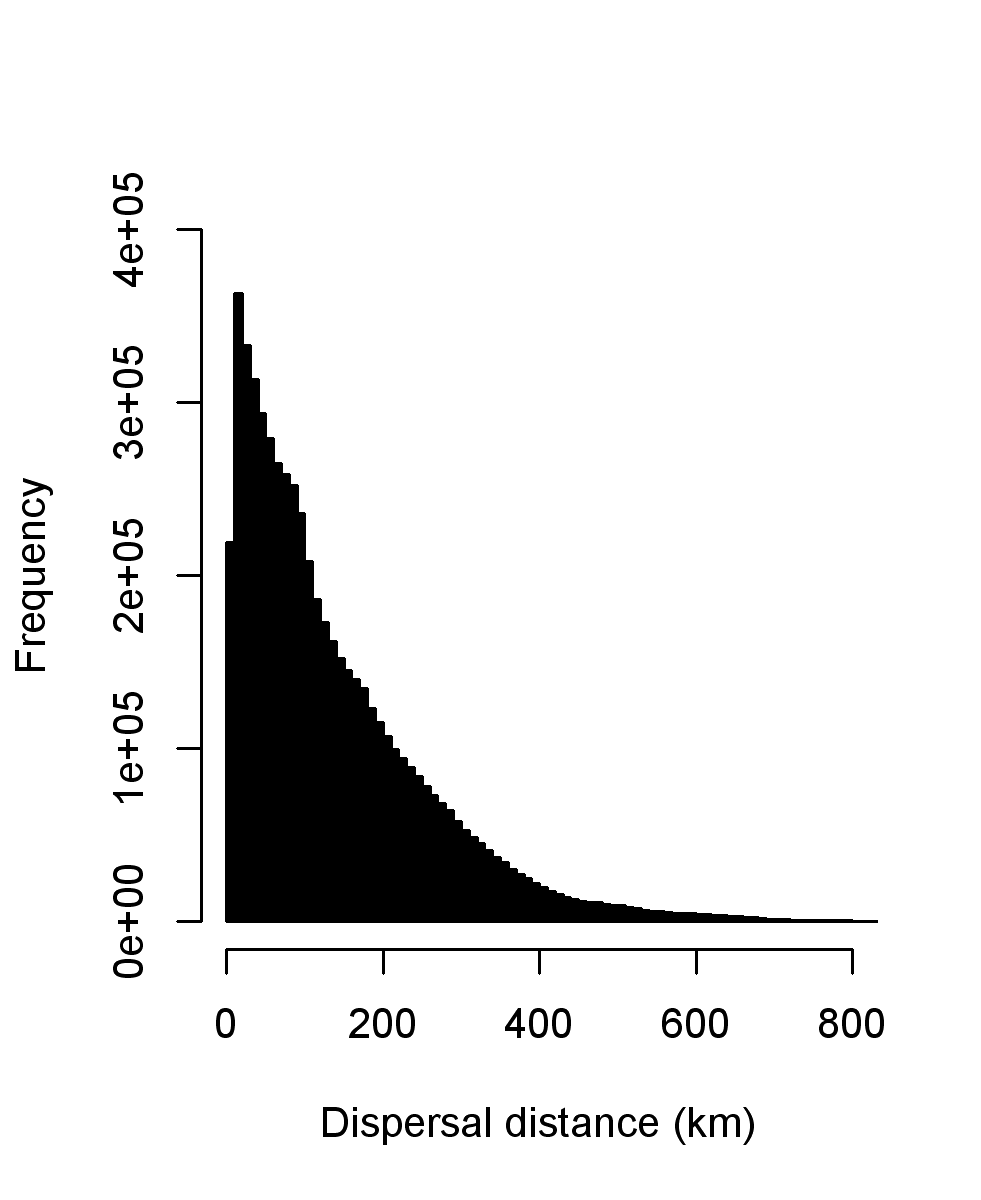

Supplement: Figure S6 — Larval dispersal distances. Frequency distribution of individual larval dispersal distances from their release point over all MPAs and years (n = 5750000). (TIFF) [file pone.0068564.s006.tiff]

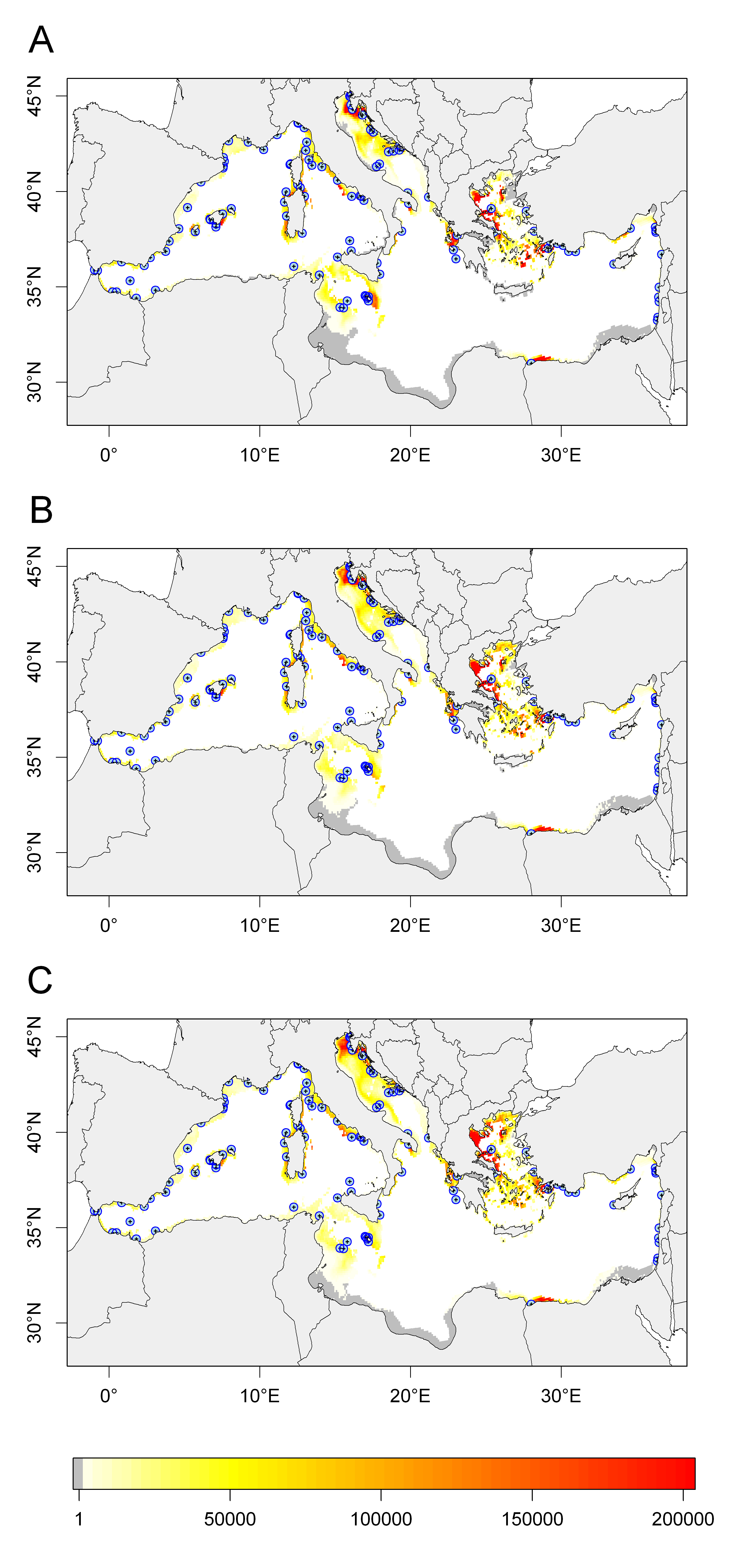

Supplement: Figure S7 — Effect of MPA larval production on larval abundance. Abundance of larvae on the continental shelf (<200 m depth) for all MPAs at the end of larval transport. MPA larval production was proportional to MPA size. A, PLD = 20 days; B, PLD = 30 days; C, PLD = 40 days. (TIFF) [file pone.0068564.s007.tiff]

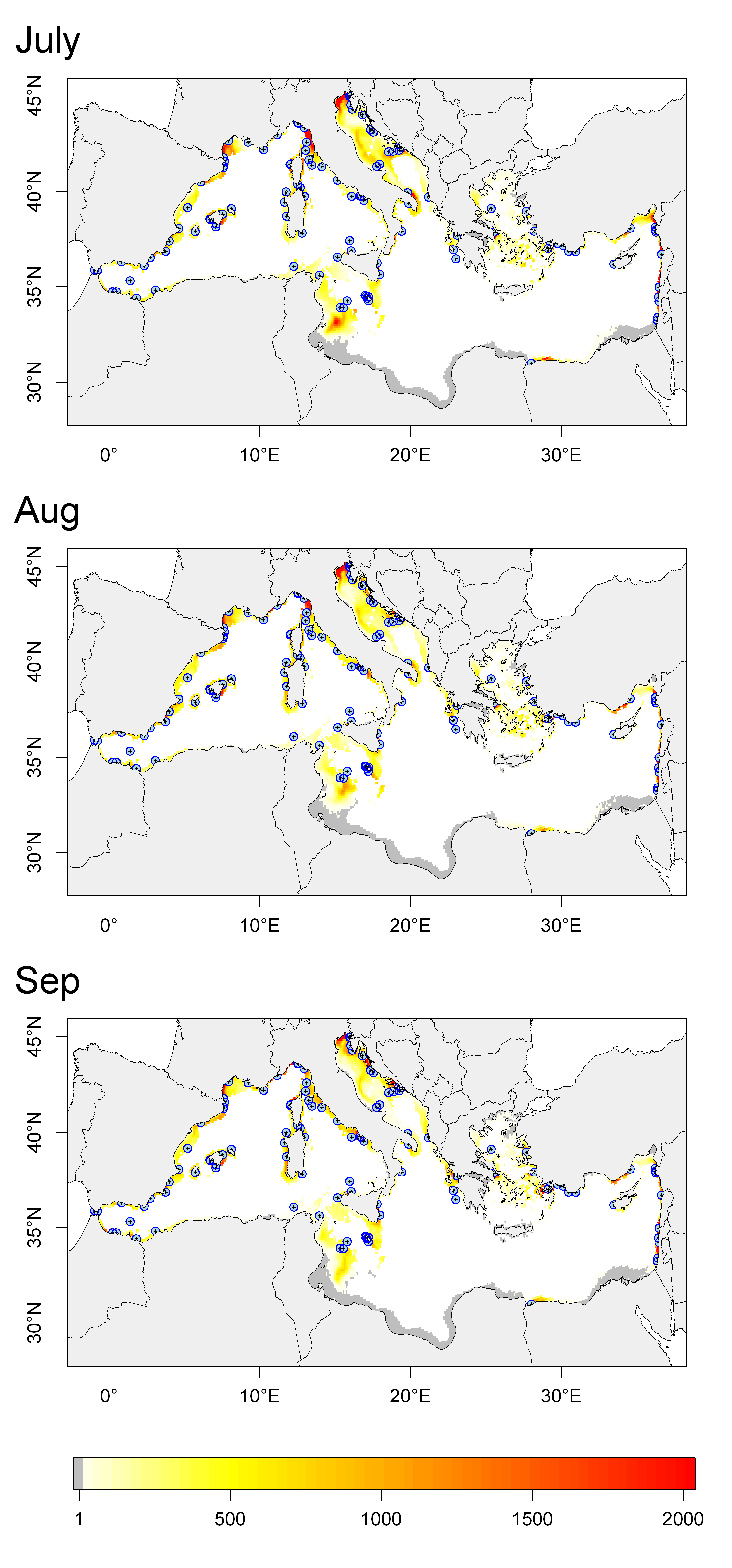

Supplement: Figure S8 — Effect of spawning month on larval abundance. Abundance of larvae on the continental shelf (<200 m depth) for all MPAs at the end of larval transport. (TIFF) [file pone.0068564.s008.tiff]

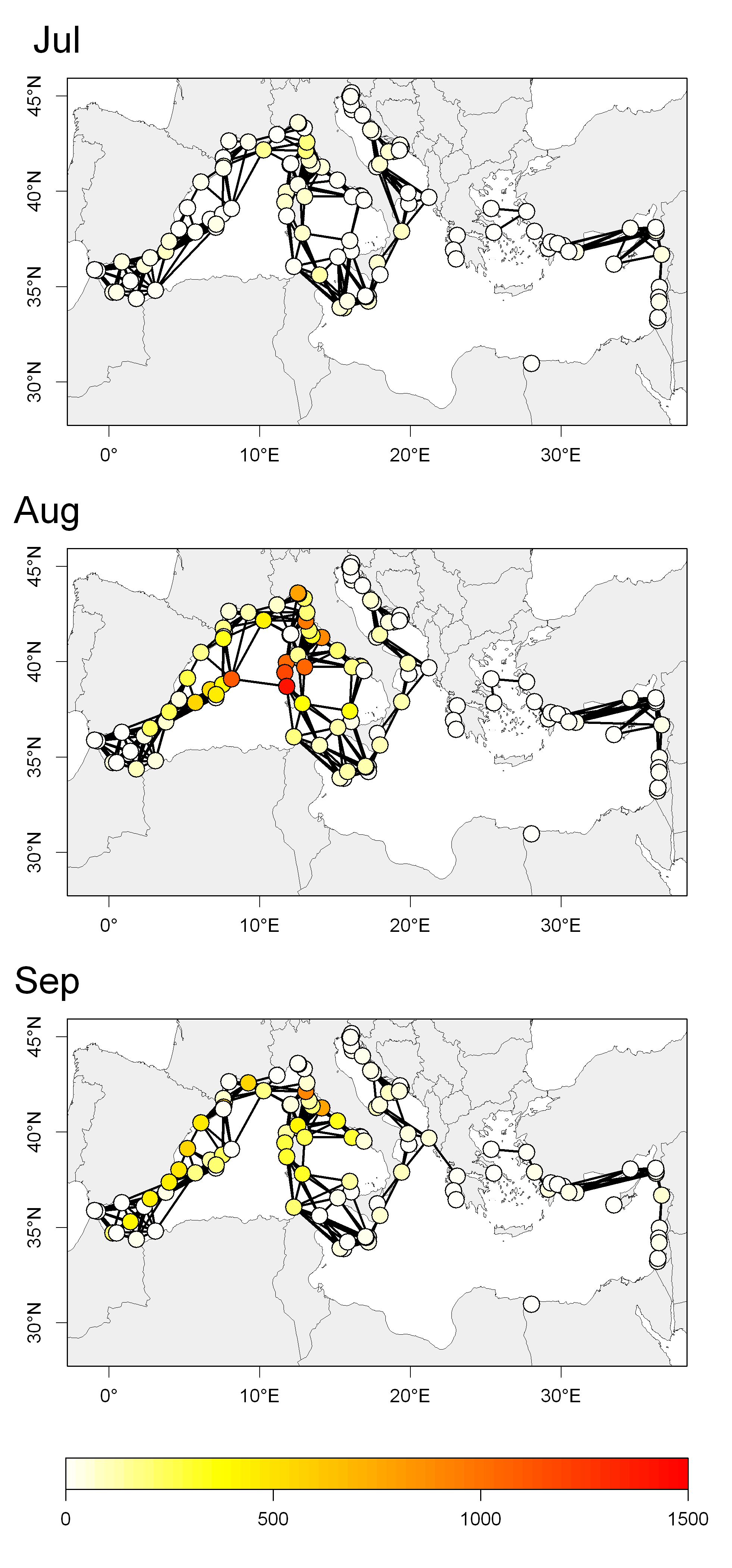

Supplement: Figure S9 — Effect of spawning month on betweenness centrality. (TIFF) [file pone.0068564.s009.tiff]

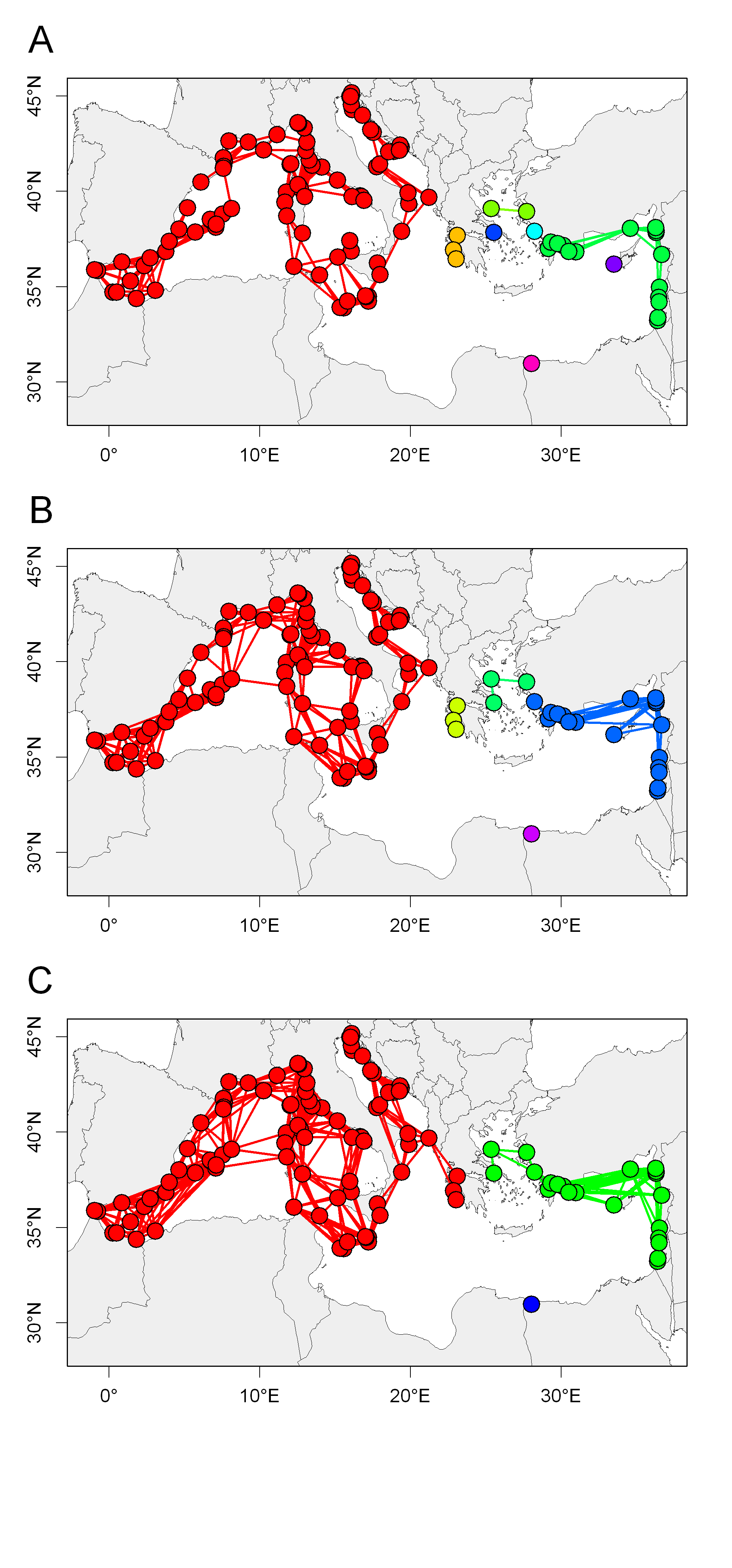

Supplement: Figure S11 — Clusters (weak connectivity). Colors represent clusters, identified using a ‘weak’ connectivity criterion (see methods). A, PLD = 20 days; B, PLD = 30 days; C, PLD = 40 days. (TIFF) [file pone.0068564.s011.tiff]
